# Supplementary material for: Rapid SARS-CoV-2 Variants Enzymatic Detection (SAVED) by CRISPR-Cas12a
Source: Microbiol Spectr. 2022 Nov 7;10(6):e03260-22. doi: 10.1128/spectrum.03260-22 (PMC9769947; doi:10.1128/spectrum.03260-22)
Supplement: Supplemental file 1 — Supplemental material. Download spectrum.03260-22-s0001.pdf, PDF file, 3.4 MB [file spectrum.03260-22-s0001.pdf]

# Rapid SARS-CoV-2 Variants Enzymatic Detection (SAVED) by CRISPR-Cas12a

## SUPPLEMENTARY MATERIALS

## Supplementary figures

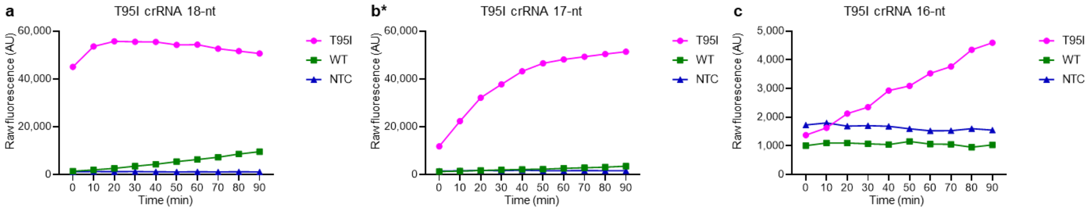


**Figure S1. T95I crRNAs fluorescence signal during 1.5 h incubation.** CRISPR-Cas12a mixture containing 100 nM Cas12a and 200 nM crRNA. **a**, T95I crRNA 18-nt. **b***, T95I crRNA 17-nt, ***** indicates the figure is the same as Fig. 1a and is included here for comparison. **c**, T95I crRNA 16-nt. CRISPR-Cas12a detection after RPA using 1E+09 copies/µl of synthetic DNA containing gene fragments of SARS-CoV-2. The 17-nt crRNA was selected for detection as the specificity of 18-nt crRNA was not acceptable, and the reaction speed of 16-nt crRNA was not adequate. AU means arbitrary units.

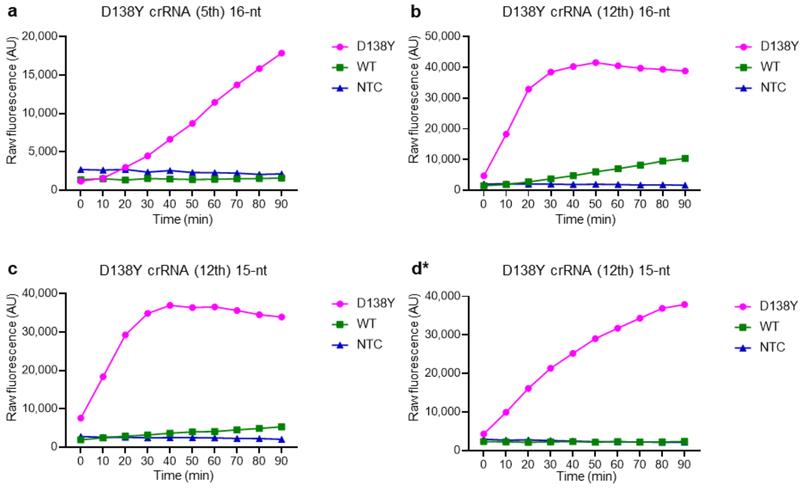


**Figure S2. D138Y crRNAs fluorescence signal during 1.5 h incubation.** CRISPR-Cas12a mixture containing 100 nM Cas12a and 200 nM crRNA except **d** with 40 nM Cas12a and 40 nM crRNA. **a**, D138Y crRNA (5th) 16-nt. **b**, D138Y crRNA (12th) 16-nt. **c**, D138Y crRNA (12th) 15-nt. **d***, D138Y crRNA (12th) 15-nt, * indicates the figure is the same as Fig. 1b and is included here for comparison. CRISPR-Cas12a detection after RPA using 1E+09 copies/µl of synthetic DNA containing gene fragments of SARS-CoV-2. (12th) 15-nt was selected based on the specificity of (12th) 15-nt, which was comparatively higher than (12th) 16-nt, and the reaction speed was faster than (5th) 16-nt at 100 nM Cas12a. To seek higher specificity of (12th) 15-nt, we used 40 nM Cas12a, which showed higher specificity. AU means arbitrary units.

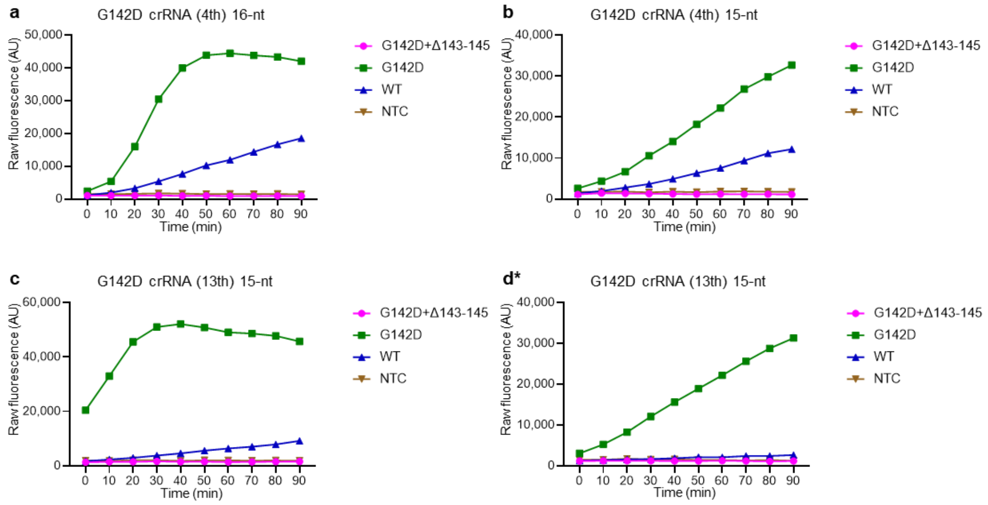


**Figure S3. G142D crRNAs fluorescence signal during 1.5 h incubation.** CRISPR-Cas12a mixture containing 100 nM Cas12a and 200 nM crRNA except **d** with 40 nM Cas12a and 40 nM crRNA. **a**, G142D crRNA (4th) 16-nt. **b**, G142D crRNA (4th) 15-nt. **c**, G142D crRNA (13th) 15-nt. **d***, G142D crRNA (13th) 15-nt, * indicates the figure is the same as Fig. 1c and is included here for comparison. CRISPR-Cas12a detection after RPA using 1E+09 copies/µl of synthetic DNA containing gene fragments of SARS-CoV-2. Among them, (13th) 15-nt was chosen as it exhibited the highest specificity and speed. We also evaluated two concentrations of Cas12a (40 nM and 100 nM) for (13th) 15-nt crRNA, and the lower concentration showed higher specificity. AU means arbitrary units.

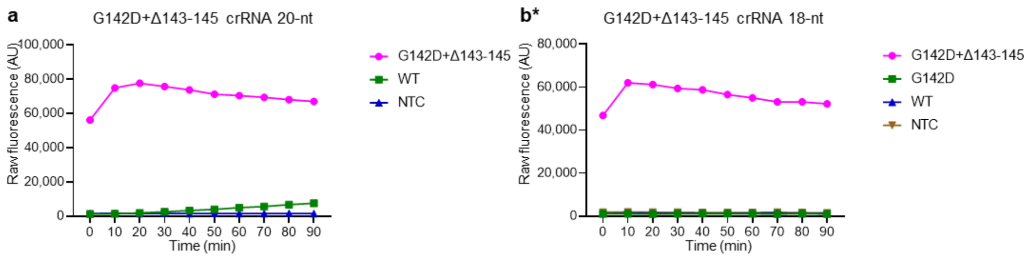


**Figure S4. G142D+Δ143-145 crRNAs fluorescence signal during 1.5 h incubation.** CRISPR-Cas12a mixture containing 100 nM Cas12a and 200 nM crRNA. **a**, G142D+Δ143-145 crRNA 20-nt. **b***, G142D+Δ143-145 crRNA 18-nt, * indicates the figure is the same as Fig. 1d and is included here for comparison. CRISPR-Cas12a detection after RPA using 1E+09 copies/µl of synthetic DNA containing gene fragments of SARS-CoV-2. Both specificity and reaction were acceptable, the 18-nt crRNA was chosen as it showed a bit higher specificity. AU means arbitrary units.

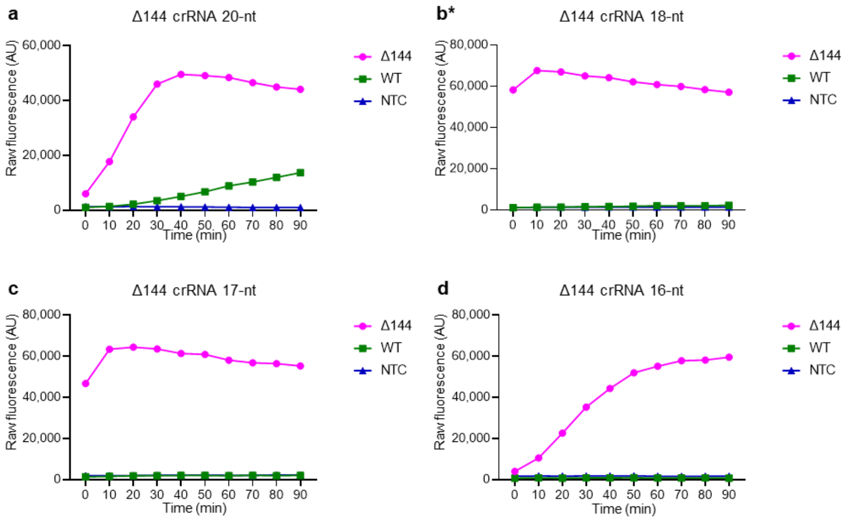


**Figure S5. Δ144 crRNAs fluorescence signal during 1.5 h incubation.** CRISPR-Cas12a mixture containing 100 nM Cas12a and 200 nM crRNA except **a** with 40 nM Cas12a and 40 nM crRNA. **a**, Δ144 crRNA 20-nt. **b***, Δ144 crRNA 18-nt, * indicates the figure is the same as Fig. 1e and is included here for comparison. **c**, Δ144 crRNA 17-nt. **d**, Δ144 crRNA 16-nt. CRISPR-Cas12a detection after RPA using 1E+09 copies/µl of synthetic DNA containing gene fragments of SARS-CoV-2. The 18-nt crRNA was chosen based on the specificity and the reaction speed. AU means arbitrary units.

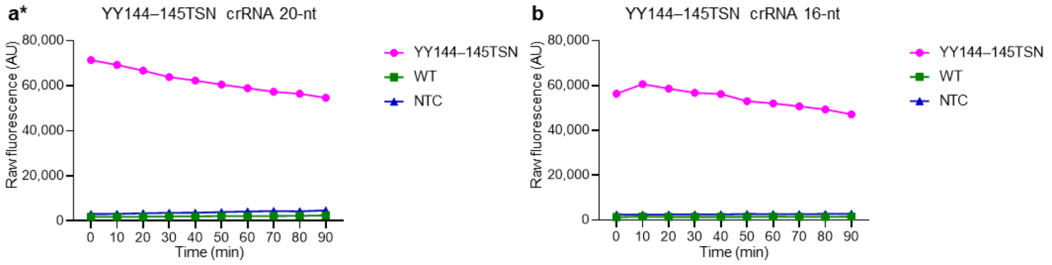


**Figure S6. YY144–145TSN crRNAs fluorescence signal during 1.5 h incubation.** CRISPR-Cas12a mixture containing 100 nM Cas12a and 200 nM crRNA. **a***, YY144–145TSN crRNA 20-nt, * indicates the figure is the same as Fig. 1f and is included here for comparison. **b**, YY144–145TSN crRNA 16-nt. CRISPR-Cas12a detection after RPA using 1E+09 copies/µl of synthetic DNA containing gene fragments of SARS-CoV-2. Both specificity and reaction speed were acceptable, the 20-nt crRNA was chosen as the reaction speed was faster. AU means arbitrary units.

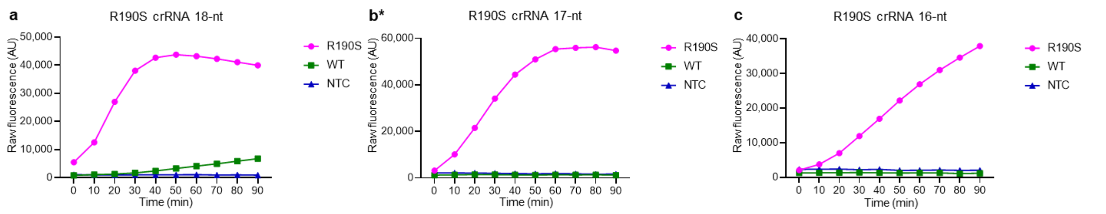


**Figure S7. R190S crRNAs fluorescence signal during 1.5 h incubation.** CRISPR-Cas12a mixture containing 100 nM Cas12a and 200 nM crRNA. **a**, R190S crRNA 18-nt. **b***, R190S crRNA 17-nt, ***** indicates the figure is the same as Fig. 1g and is included here for comparison. **c**, R190S crRNA 16-nt. CRISPR-Cas12a detection after RPA using 1E+09 copies/μl of synthetic DNA containing gene fragments of SARS-CoV-2. Based on the reaction speed, 20-nt crRNA was selected for further study. The 17-nt crRNA was chosen based on the speed of the reaction as the specificity of both 17-nt and 16-nt were acceptable. AU means arbitrary units.

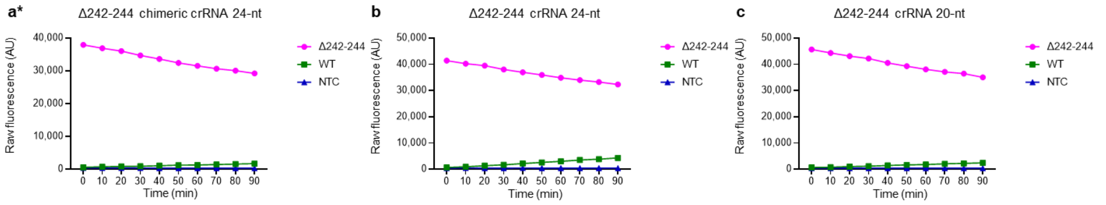


**Figure S8. Δ242-244 crRNAs fluorescence signal during 1.5 h incubation.** CRISPR-Cas12a mixture containing 40 nM Cas12a and 40 nM crRNA. **a***, Δ242-244 chimeric crRNA 24-nt, ***** indicates the figure is the same as Fig. 1i and is included here for comparison. **b**, Δ242-244 crRNA 24-nt. **c**, Δ242-244 crRNA 20-nt. CRISPR-Cas12a detection after RPA using 1E+09 copies/µl of synthetic DNA containing gene fragments of SARS-CoV-2. The specificity and reaction speed of all three crRNAs were acceptable. The chimeric 24-nt was chosen as the WT signal was the weakest in comparison after 1.5 h incubation. AU means arbitrary units.

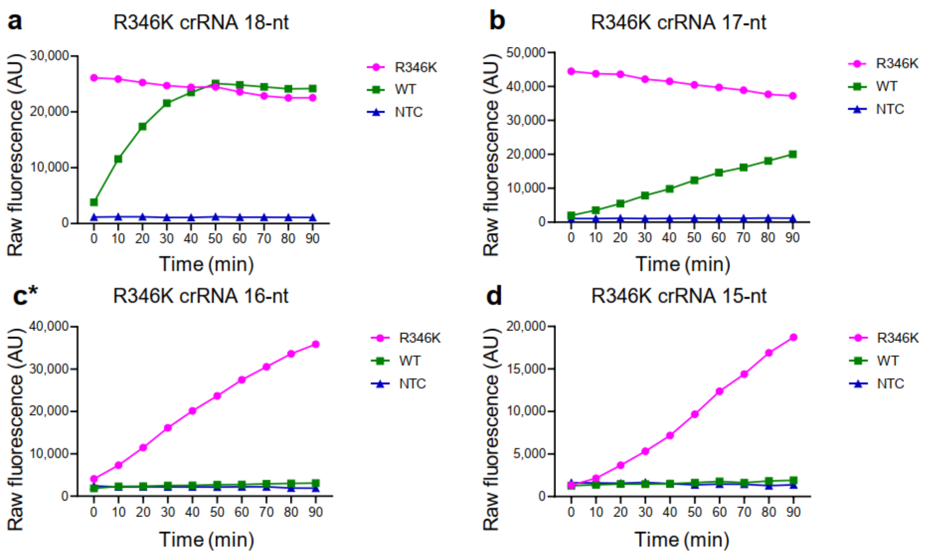


**Figure S9. R346K crRNAs fluorescence signal during 1.5 h incubation.** CRISPR-Cas12a mixture containing 100 nM Cas12a and 200 nM crRNA. **a**, R346K crRNA 18-nt. **b**, R346K crRNA 17-nt. **c***, R346K crRNA 16-nt, ***** indicates the figure is the same as Fig. 1j and is included here for comparison. **d**, R346K crRNA 15-nt. CRISPR-Cas12a detection after RPA using 1E+09 copies/µl of synthetic DNA containing gene fragments of SARS-CoV-2. The specificity and reaction speed of 16-nt and 15-nt were acceptable. The 16-nt crRNA was selected for further study as it reacted faster than 15-nt. AU means arbitrary units.

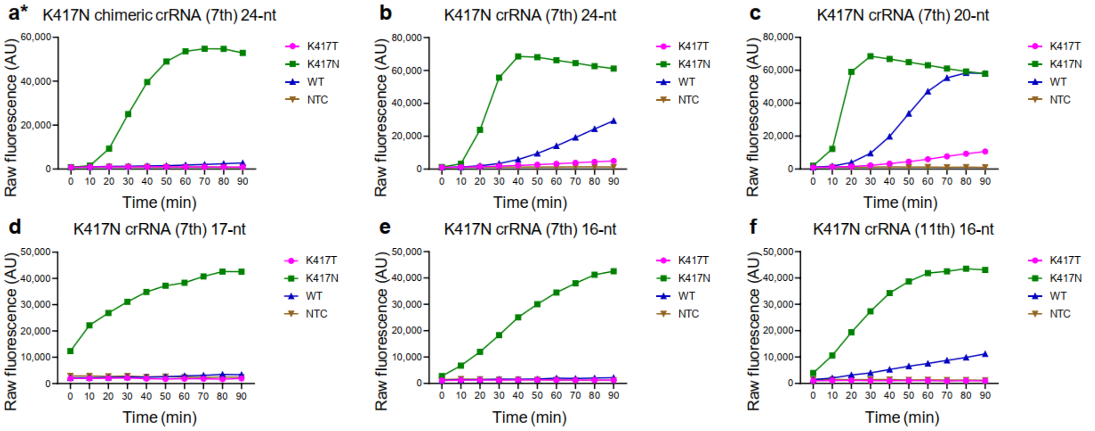


**Figure S10. K417N crRNAs fluorescence signal during 1.5 h incubation.** CRISPR-Cas12a mixture containing 40 nM Cas12a and 40 nM crRNA. **a***, K417N chimeric crRNA (7th) 24-nt, * indicates the figure is the same as Fig. 1k and is included here for comparison. **b**, K417N crRNA (7th) 24-nt. **c**, K417N crRNA (7th) 20-nt. **d**, K417N crRNA (7th) 17-nt. **e**, K417N crRNA (7th) 16-nt. **f**, K417N crRNA (11th) 16-nt. CRISPR-Cas12a detection after RPA using 1E+09 copies/µl of synthetic DNA containing gene fragments of SARS-CoV-2. The specificity of chimeric (7th) 24-nt, (7th) 17-nt (WT signal increased but not much), and (7th) 16-nt were acceptable, and we selected the chimeric (7th) 24-nt based on reaction speed and specificity. AU means arbitrary units.

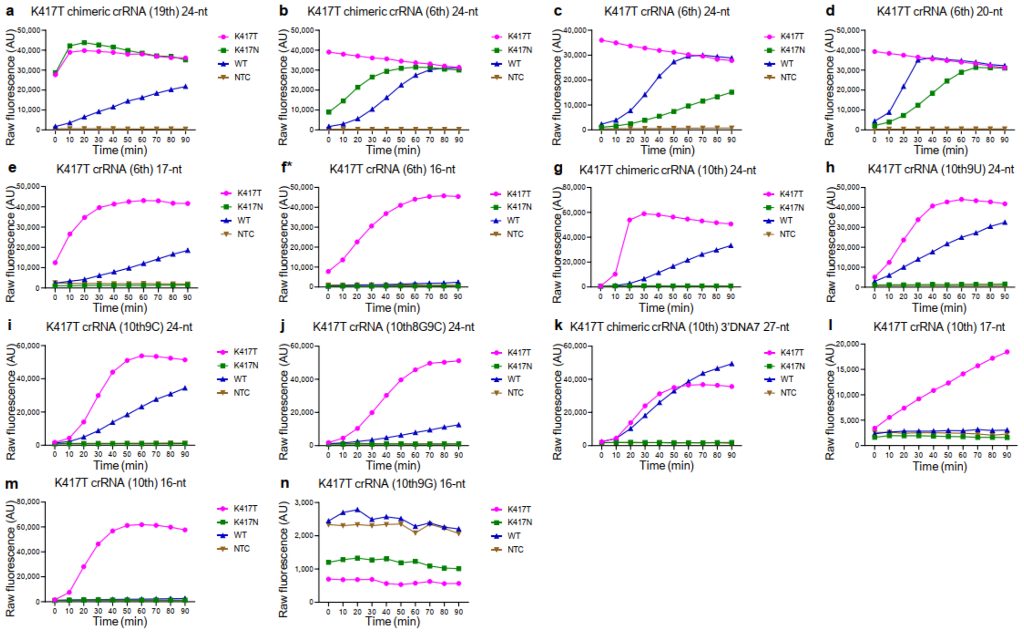


**Figure S11. K417T crRNAs fluorescence signal during 1.5 h incubation.** CRISPR-Cas12a mixture containing 40 nM Cas12a and 40 nM crRNA. **a**, K417T chimeric crRNA (19th) 24-nt. **b**, K417T chimeric crRNA (6th) 24-nt. **c**, K417T crRNA (6th) 24-nt. **d**, K417T crRNA (6th) 20-nt. **e**, K417T crRNA (6th) 17-nt. **f***, K417T crRNA (6th) 16-nt, * indicates the figure is the same as Fig. 1l and is included here for comparison. **g**, K417T chimeric crRNA (10th) 24-nt. **h**, K417T crRNA (10th9U) 24-nt. **i**, K417T crRNA (10th9C) 24-nt. **j**, K417T crRNA (10th8G9C) 24-nt. **k**, K417T chimeric crRNA (10th) 3’DNA7 27-nt (20-nt spacer+3’DNA7). **l**, K417T crRNA (10th) 16-nt. **m**, K417T crRNA (10th) 16-nt. **n**, K417T crRNA (10th9G) 16-nt. CRISPR-Cas12a detection after RPA using 1E+09 copies/µl of synthetic DNA containing gene fragments of SARS-CoV-2. The (6th) 16-nt, (10th) 17-nt crRNA, and (10th) 16-nt crRNA specificity were acceptable, and (6th) 16-nt was chosen as it requires the same RT-RPA primers as used in K417N detection. AU means arbitrary units.

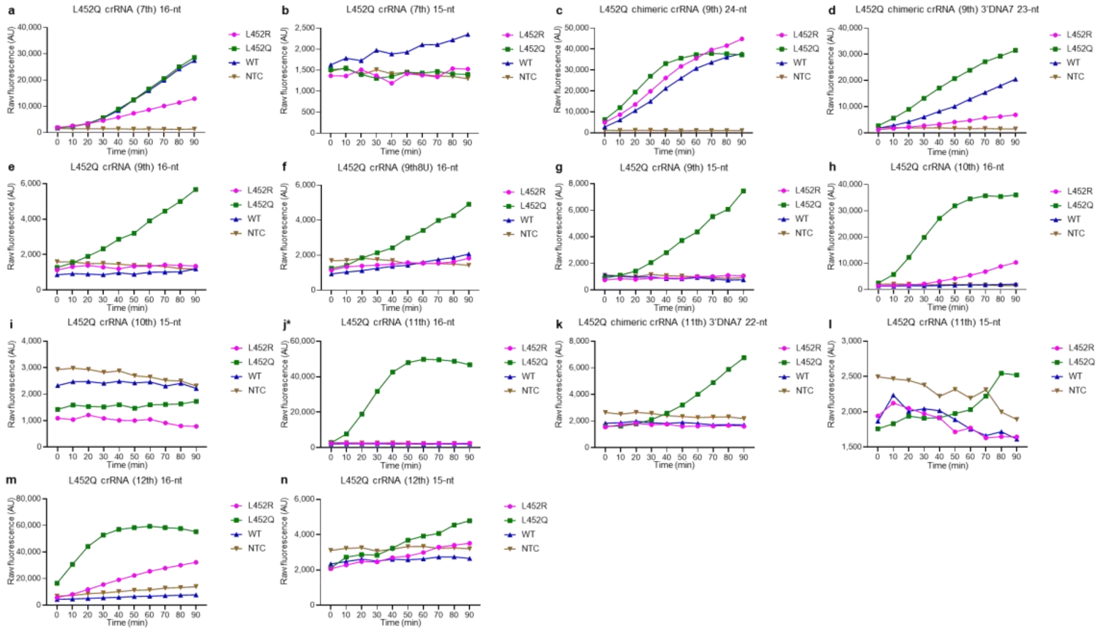


**Figure S12. L452Q crRNAs fluorescence signal during 1.5 h incubation.** CRISPR-Cas12a mixture containing 100 nM Cas12a and 200 nM crRNA except **c-g** with 40 nM Cas12a and 40 nM crRNA. **a**, L452Q crRNA (7th) 16-nt. **b**, L452Q crRNA (7th) 15-nt. **c**, L452Q chimeric crRNA (9th) 24-nt. **d**, L452Q chimeric crRNA (9th) 3'DNA7 23-nt (16-nt spacer+3'DNA7). **e**, L452Q crRNA (9th) 16-nt. **f**, L452Q crRNA (9th8U) 16-nt. **g**, L452Q crRNA (9th) 15-nt. **h**, L452Q crRNA (10th) 16-nt. **i**, L452Q crRNA (10th) 15-nt. **j***, L452Q crRNA (11th) 16-nt, * indicates the figure is the same as Fig. 1m and is included here for comparison. **k**, L452Q chimeric crRNA (11th) 3'DNA7 22-nt (15-nt spacer+3'DNA7). **l**, L452Q crRNA (11th) 15-nt. **m**, L452Q crRNA (12th) 16-nt. **n**, L452Q crRNA (12th) 15-nt. CRISPR-Cas12a detection after RPA using 1E+09 copies/µl of synthetic DNA containing gene fragments of SARS-CoV-2. Based on the specificity and reaction speed, only (11th) 16-nt was acceptable. AU means arbitrary units.

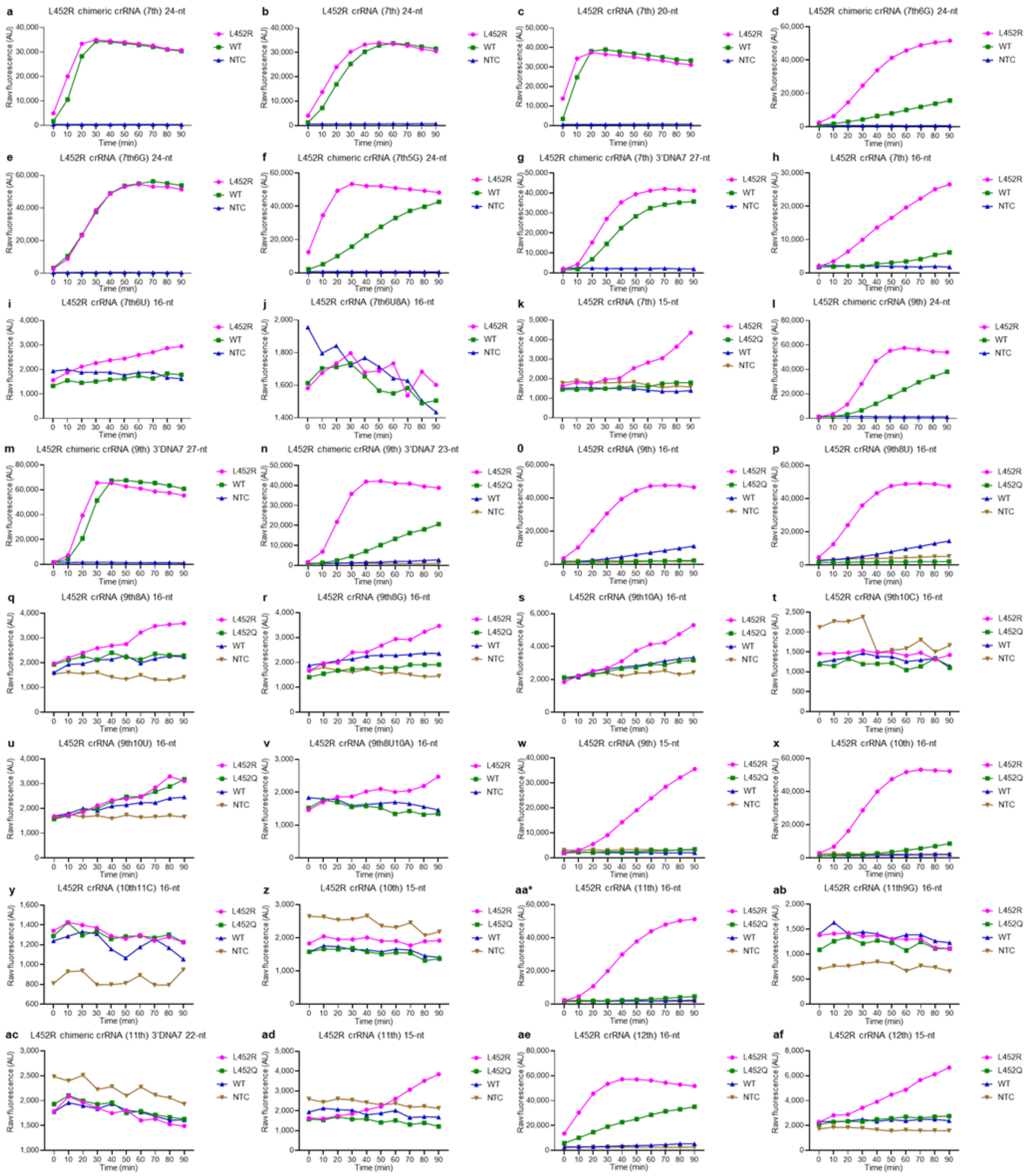


**Figure S13. L452R crRNAs fluorescence signal during 1.5 h incubation.** CRISPR-Cas12a mixture containing with 40 nM Cas12a and 40 nM crRNA except **l-af** with 100 nM Cas12a and 200 nM crRNA. **a**, L452R chimeric crRNA (7th) 24-nt. **b**, L452R crRNA (7th) 24-nt. **c**, L452R crRNA (7th) 20-nt. **d**, L452R chimeric crRNA (7th6G) 24-nt. **e**, L452R crRNA (7th6G) 24-nt. **f**, L452R chimeric crRNA (7th5G) 24-nt. **g**, L452R chimeric crRNA (7th) 3'DNA7 27-nt (20-nt spacer+3'DNA7). **h**, L452R crRNA (7th) 16-nt. **i**, L452R crRNA (7th6U) 16-nt. **j**, L452R crRNA (7th6U8A) 16-nt. **k**, L452R crRNA (7th) 15-nt. **l**, L452R chimeric crRNA (9th) 24-nt. **m**, L452R chimeric crRNA (9th) 3'DNA7 27-nt (20-nt spacer+3'DNA7). **n**, L452R chimeric crRNA (9th) 3'DNA7 23-nt (16-nt spacer+3'DNA7). **o**, L452R crRNA (9th) 16-nt. **p**, L452R crRNA (9th8U). **q**, L452R crRNA (9th8A) 16-nt. **r**, L452R crRNA (9th8G)

16-nt. **s**, L452R crRNA (9th10A) 16-nt. **t**, L452R crRNA (9th10C) 16-nt. **u**, L452R crRNA (9th10U) 16-nt. **v**, L452R crRNA (9th8U10A) 16-nt. **w**, L452R crRNA (9th) 15-nt. **x**, L452R crRNA (10th) 16-nt. **y**, L452R crRNA (10th11C) 16-nt. **z**, L452R crRNA (10th) 15-nt. **aa***, L452R crRNA (11th) 16-nt, * indicates the figure is the same as Fig. 1n and is included here for comparison. **ab**, L452R crRNA (11th9G) 16-nt. **ac**, L452R chimeric crRNA (11th) 3'DNA7 22-nt. **ad**, L452R crRNA (11th) 15-nt. **ae**, L452R crRNA (12th) 16-nt. **af**, L452R crRNA (12th) 15-nt. CRISPR-Cas12a detection after RPA using 1E+09 copies/µl of synthetic DNA containing gene fragments of SARS-CoV-2. The (11th) 16-nt was selected as only its specificity and reaction speed were acceptable. AU means arbitrary units.

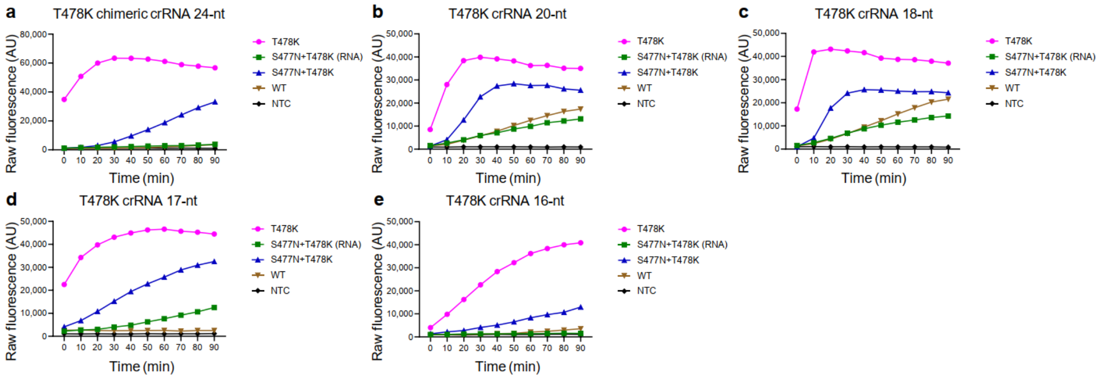


**Figure S14. T478K crRNAs fluorescence signal during 1.5 h incubation.** CRISPR-Cas12a mixture containing 100 nM Cas12a and 200 nM. **a**, T478K chimeric crRNA 24-nt. **b**, T478K crRNA 20-nt. **c**, T478K crRNA 18-nt. **d**, T478K crRNA 17-nt. **e**, T478K crRNA 16-nt. T478K RT-RPA primers were used for RPA or RT-RPA using 1E+09 copies/µl of synthetic DNA/RNA containing gene fragments of SARS-CoV-2 for CRISPR-Cas12a detection. Omicron BA.2 was used as template of S477N+T478K, which has two mismatches in the reverse primer compared with Delta variant. Chimeric 24-nt crRNA was selected based on the speed of the reaction and specificity. AU means arbitrary units.

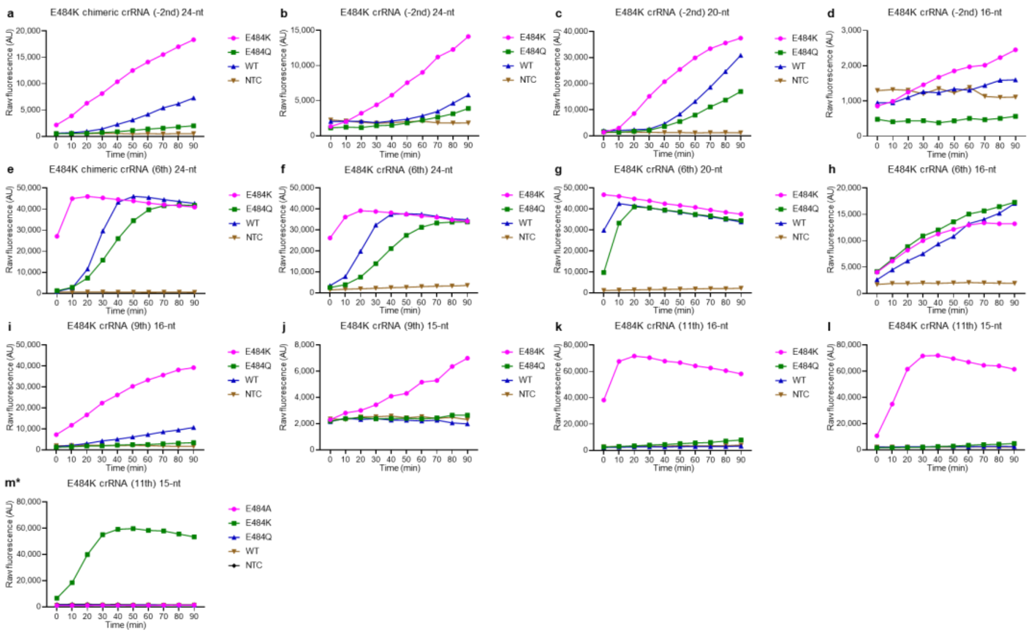


**Figure S15. E484K crRNAs fluorescence signal during 1.5 h incubation.** CRISPR-Cas12a mixture containing 40 nM Cas12a and 40 nM crRNA except **i-m** with 100 nM Cas12a and 200 nM crRNA. **a**, E484K chimeric crRNA (-2nd) 24-nt. **b**, E484K crRNA (-2nd) 24-nt. **c**, E484K crRNA (-2nd) 20-nt. **d**, E484K crRNA (-2nd) 16-nt. **e**, E484K chimeric crRNA (6th) 24-nt. **f**, E484K crRNA (6th) 24-nt. **g**, E484K crRNA (6th) 20-nt. **h**, E484K crRNA (6th) 16-nt. **i**, E484K crRNA (9th) 16-nt. **j**, E484K crRNA (9th) 15-nt. **k**, E484K crRNA (11th) 16-nt. **l**, E484K crRNA (11th) 15-nt. **m***, E484K crRNA (11th) 15-nt, ***** indicates the figure is the same as Fig. 1p and is included here for comparison. CRISPR-Cas12a detection after RPA using 1E+09 copies/µl of synthetic DNA containing gene fragments of SARS-CoV-2. The (11th) 15-nt was selected as the specificity was relatively higher than (11th) 16-nt, and the reaction speed was comparatively faster than (9th) 15-nt. AU means arbitrary units.

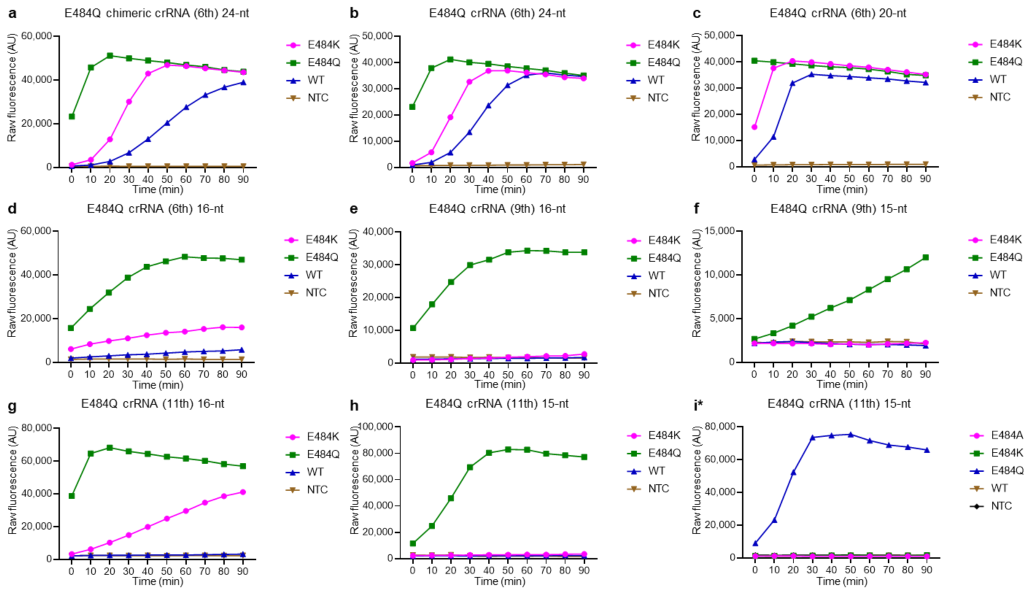


**Figure S16. E484Q crRNAs fluorescence signal during 1.5 h incubation.** CRISPR-Cas12a mixture containing 40 nM Cas12a and 40 nM crRNA except **e-i** with 100 nM Cas12a and 200 nM crRNA. **a**, E484Q chimeric crRNA (6th) 24-nt. **b**, E484Q crRNA (6th) 24-nt. **c**, E484Q crRNA (6th) 20-nt. **d**, E484Q crRNA (6th) 16-nt. **e**, E484Q crRNA (9th) 16-nt. **f**, E484Q crRNA (9th) 15-nt. **g**, E484Q crRNA (11th) 16-nt. **h**, E484Q crRNA (11th) 15-nt. **i***, E484Q crRNA (11th) 15-nt, ***** indicates the figure is the same as Fig. 1q and is included here for easy comparison. CRISPR-Cas12a detection after RPA using 1E+09 copies/µl of synthetic DNA containing gene fragments of SARS-CoV-2. The specificity of (9th) 16-nt, (9th) 15-nt, and (11th) 15-nt were acceptable. E484Q crRNA (11th) 15-nt was chosen as it shares the same RT-RPA primers as E484K. AU means arbitrary units.

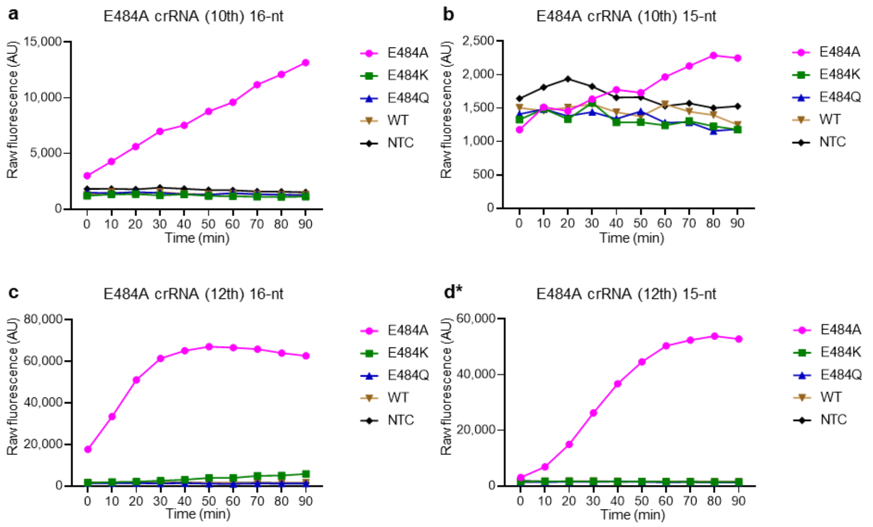


**Figure S17. E484A crRNAs fluorescence signal during 1.5 h incubation.** CRISPR-Cas12a mixture containing 100 nM Cas12a and 200 nM crRNA. **a**, E484A crRNA (10th) 16-nt. **b**, E484A crRNA (10th) 15-nt. **c**, E484A crRNA (12th) 16-nt. **d***, E484A crRNA (12th) 15-nt, * indicates the figure is the same as Fig. 1r and is included here for comparison. CRISPR-Cas12a detection after RPA using 1E+09 copies/µl of synthetic DNA containing gene fragments of SARS-CoV-2. The (12th) 15-nt crRNA was selected for further study because the specificity of (12th) 15-nt was comparatively higher than (12th) 16-nt, and the relative speed was faster than (10th) 16-nt and (10th) 15-nt. AU means arbitrary units.

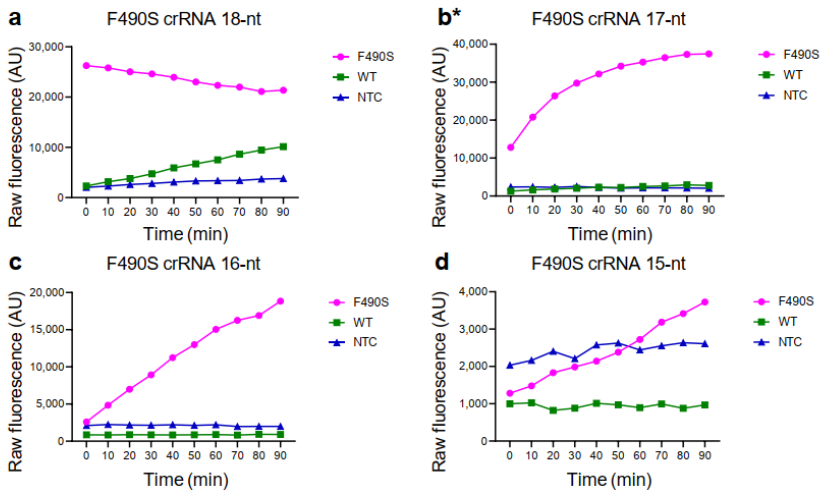


**Figure S18. F490S crRNAs fluorescence signal during 1.5 h incubation.** CRISPR-Cas12a mixture containing 100 nM Cas12a and 200 nM crRNA. **a**, F490S crRNA 18-nt. **b**, F490S crRNA 17-nt, ***** indicates the figure is the same as Fig. 1s and is included here for comparison. **c**, F490S crRNA 16-nt. **d**, F490S crRNA 15-nt. CRISPR-Cas12a detection after RPA using 1E+09 copies/µl of synthetic DNA containing gene fragments of SARS-CoV-2. Among them, 17-nt and 16-nt crRNAs' specificity were acceptable, and the 17-nt was chosen as it reacted comparatively faster. AU means arbitrary units.

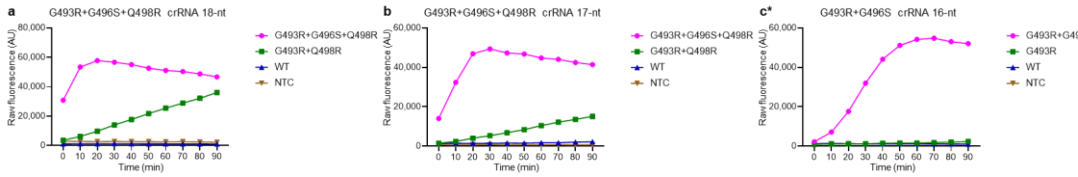


**Figure S19. G493R+G496S w/o Q498R crRNAs fluorescence signal during 1.5 h incubation.** CRISPR-Cas12a mixture containing 100 nM Cas12a and 200 nM crRNA. **a**, G493R+G496S+Q498R crRNA 18-nt. **b**, G493R+G496S+Q498R crRNA 17-nt. **c***, G493R+G496S crRNA 16-nt, ***** indicates the figure is the same as Fig. 1t and is included here for comparison. CRISPR-Cas12a detection after RPA using 1E+09 copies/µl of synthetic DNA containing gene fragments of SARS-CoV-2. Only the specificity of G493R+G496S crRNA 16-nt was acceptable, thus, it was chosen for detection. AU means arbitrary units.

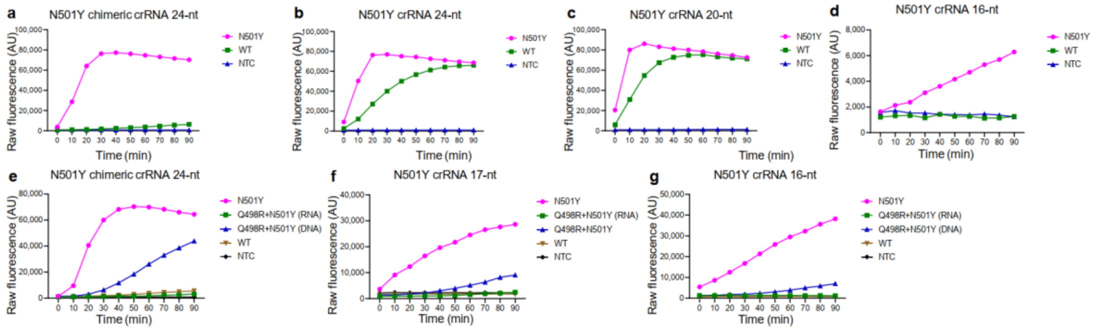


**Figure S20. N501Y crRNAs fluorescence signal during 1.5 h incubation.** CRISPR-Cas12a mixture containing 40 nM Cas12a and 40 nM crRNA except **e-g** with 100 nM Cas12a and 200 nM crRNA. **a**, N501Y chimeric crRNA 24-nt. **b**, N501Y crRNA 24-nt. **c**, N501Y crRNA 20nt. **d**, N501Y crRNA 16-nt. **e**, N501Y chimeric crRNA 24-nt with Q498R+N501Y RNA and DNA samples. **f**, N501Y crRNA 17-nt with Q498R+N501Y RNA and DNA samples. **g**, N501Y crRNA 16-nt with Q498R+N501Y RNA and DNA samples. N501Y RT-RPA primers were used for RPA or RT-RPA using 1E+09 copies/µl of synthetic DNA/RNA containing gene fragments of SARS-CoV-2 for CRISPR-Cas12a detection. N501Y chimeric crRNA 24-nt was selected based on the reaction speed and specificity. AU means arbitrary units.

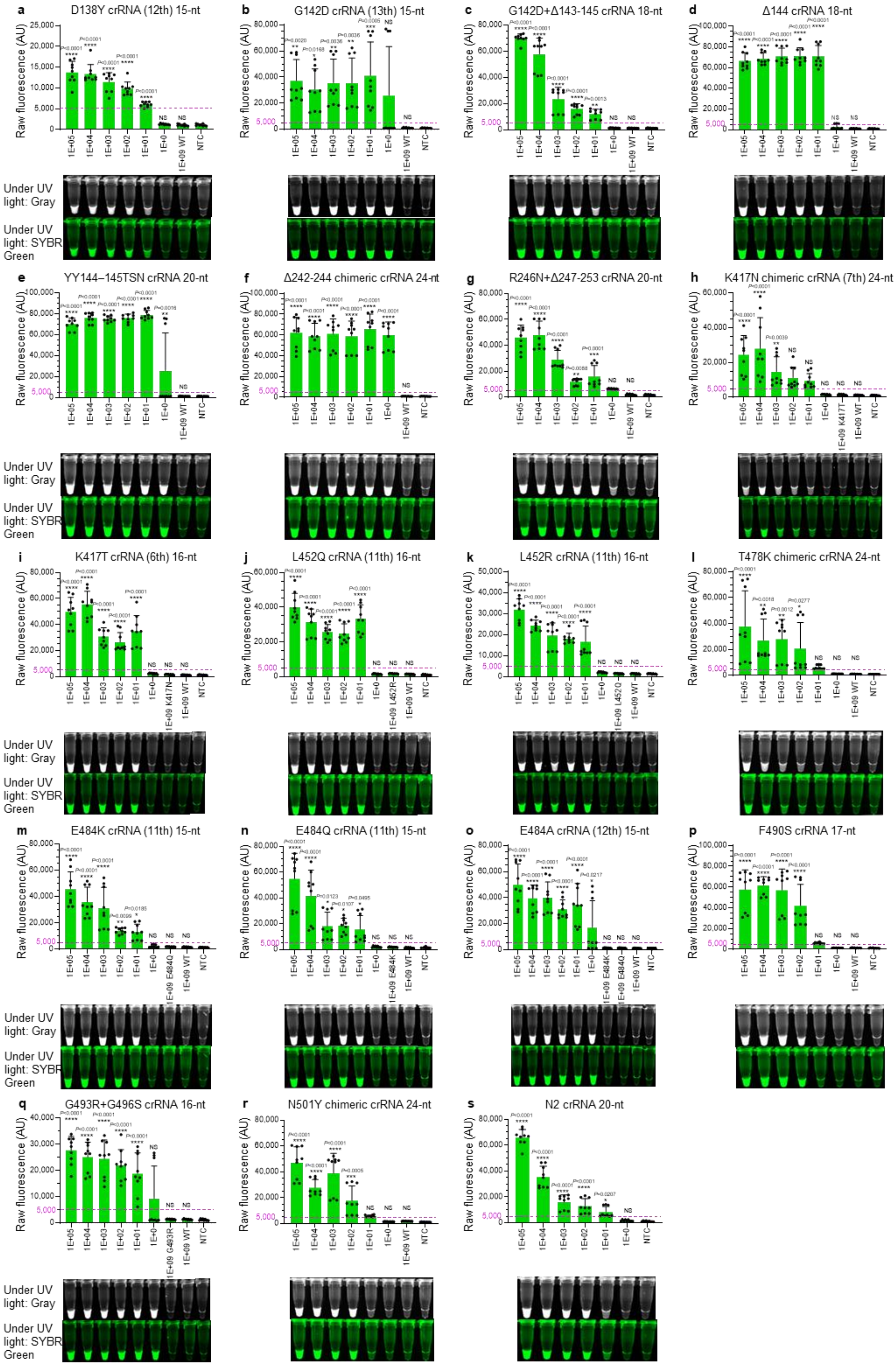


**Figure S21. LoD and specificity of fluorescence and UV light detection using**

**serial dilutions of SARS-CoV-2 variants IVT RNA after incubation at 37°C for 30 min.** CRISPR-Cas12a mixture containing 100 nM Cas12a and 200 nM crRNA except **a**, **b**, **f**, and **g** with 40 nM Cas12a and 40 nM crRNA. **a**, D138Y crRNA (12th) 15-nt. **b**, G142D crRNA (13th) 15-nt. **c**, G142D+Δ143-145 crRNA 18-nt. **d**, Δ144 crRNA 18-nt. **e**, YY144–145TSN crRNA 20-nt. **f**, Δ242-244 chimeric crRNA 24-nt. **g**, R246N+Δ247-253 crRNA 20-nt. **h**, K417N chimeric crRNA (7th) 24-nt. **i**, K417T crRNA (6th) 16-nt. **j**, L452Q crRNA (11th) 16-nt. **k**, L452R crRNA (11th) 16-nt. **l**, T478K chimeric crRNA 24-nt. **m**, E484K crRNA (11th) 15-nt. **n**, E484Q crRNA (11th) 15-nt. **o**, E484A crRNA (12th) 15-nt. **p**, F490S crRNA 17-nt. **q**, G493R+G496S crRNA 16-nt. **r**, N501Y chimeric crRNA 24-nt. **s**, N2 crRNA 20-nt. RT-RPA using indicated concentration of IVT RNA containing gene fragments of SARS-CoV-2 variants. NTC stands for non-template control. AU means arbitrary units. Statistical analysis was performed using a one-way ANOVA test with Dunnett's multiple comparisons test. The raw fluorescence (AU) of each reaction was compared to the respective NTCs. The horizontal pink dash line indicates the fluorescence threshold (5000 AU) of positive samples, which can be visualized under UV light. Error bars represent the mean ± standard deviation (SD) from triplicate measurements, each with three technical replicates (n=9), and each dot represents one replicate. The result under UV light with the lowest LoD was selected from triplicate measurements without technical replicate (n=3). The asterisks (*, **, ***, ****) indicate significant differences with $P < 0.05$, $P < 0.01$, $P < 0.001$, and $P < 0.0001$ and NS denotes not significant ($p > 0.05$).

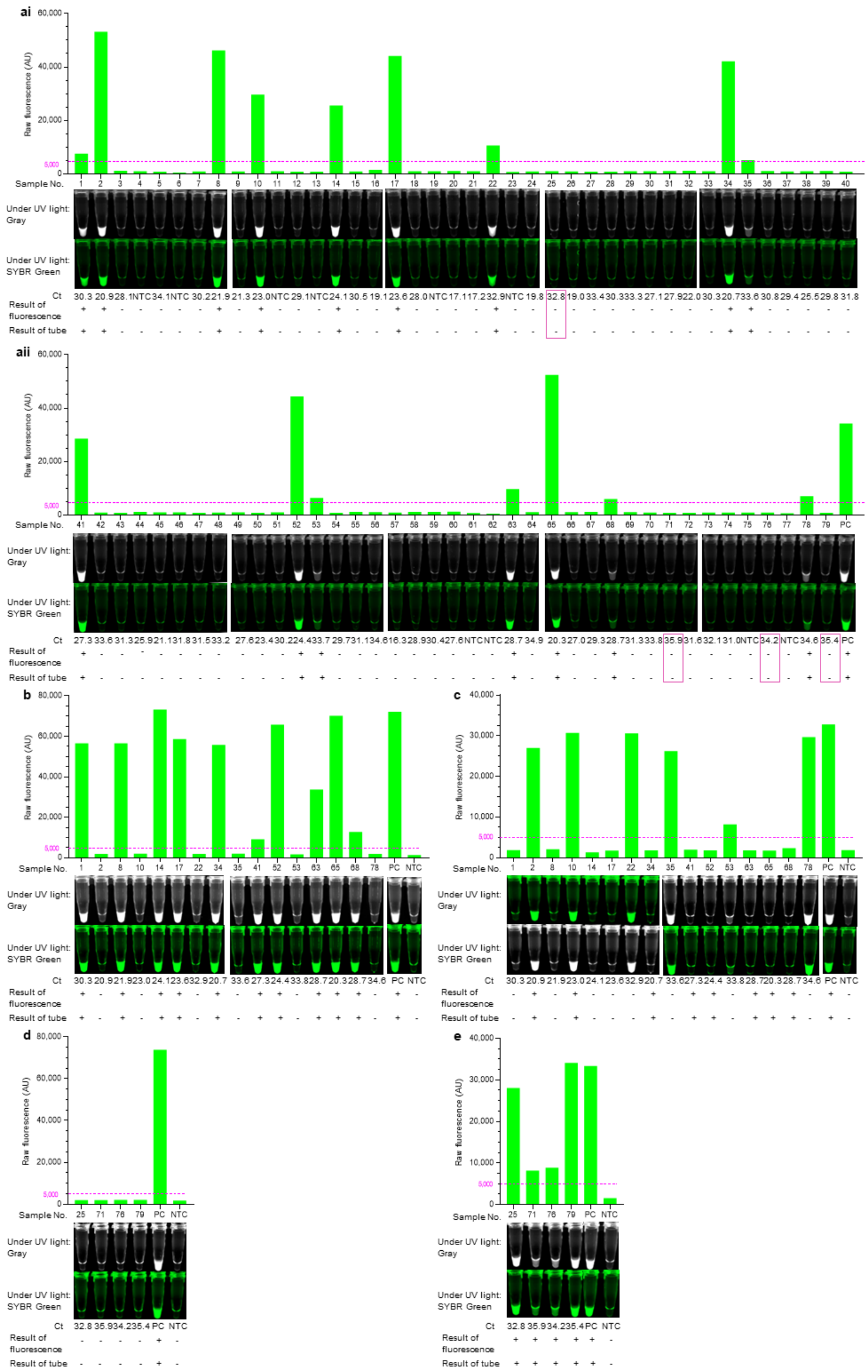


**Figure S22. Fluorescence and tube results after 30 min incubation of SAVED**

**CRISPR-Cas12a based detection of SARS-CoV-2 Alpha and Beta variants in blinded 79 samples, including 69 clinical samples and 10 NTC. ai, aii**, N501Y detection (Marker of Alpha, Beta, Gamma, and Mu. Omicron variant is not positive due to the mismatch present in the N501Y RPA F primer) in 79 samples, 16 out of 20 were positive (Ct range: 20.3-33.7), 4 in 20 were false negative (Ct range: 32.8-35.9), no false positive (100% specificity, Ct: 16.3-34.9), so the sensitivity was 80% (Ct range: 20.3-35.9), but for samples Ct < 32.8 (≤30.3) showed 100% sensitivity. **b**, Δ144 detection (Alpha marker) in 16 N501Y positive samples, 10 were positive (Ct range: 20.3-30.3), with no false positive or negative. **c**, Δ242-244 detection (Beta marker) ) in 16 N501Y positive samples, 6 of them were positive, no false positive or negative (Ct range: 20.9-33.7). **d**, Δ144 detection (Alpha marker) in 4 N501Y false-negative samples, none is positive, no false negative. **e**, Δ242-244 detection (Beta marker) ) in 4 N501Y false-negative samples, all are positive, no false positive (Ct range: 32.8-35.9). So the Δ144 detection with 100% sensitivity (Ct range: 20.3-30.3) and 100% specificity (Ct range: 20.9-35.9), the Δ242-244 detection with 100% sensitivity (Ct range: 20.9-35.9) and 100% specificity (Ct range: 20.3-30.3). AU means arbitrary units. The horizontal pink dash line indicates the fluorescence threshold (5000 AU) of positive samples, which can be visualized under UV light. False-negative samples are highlighted by pink rectangles.

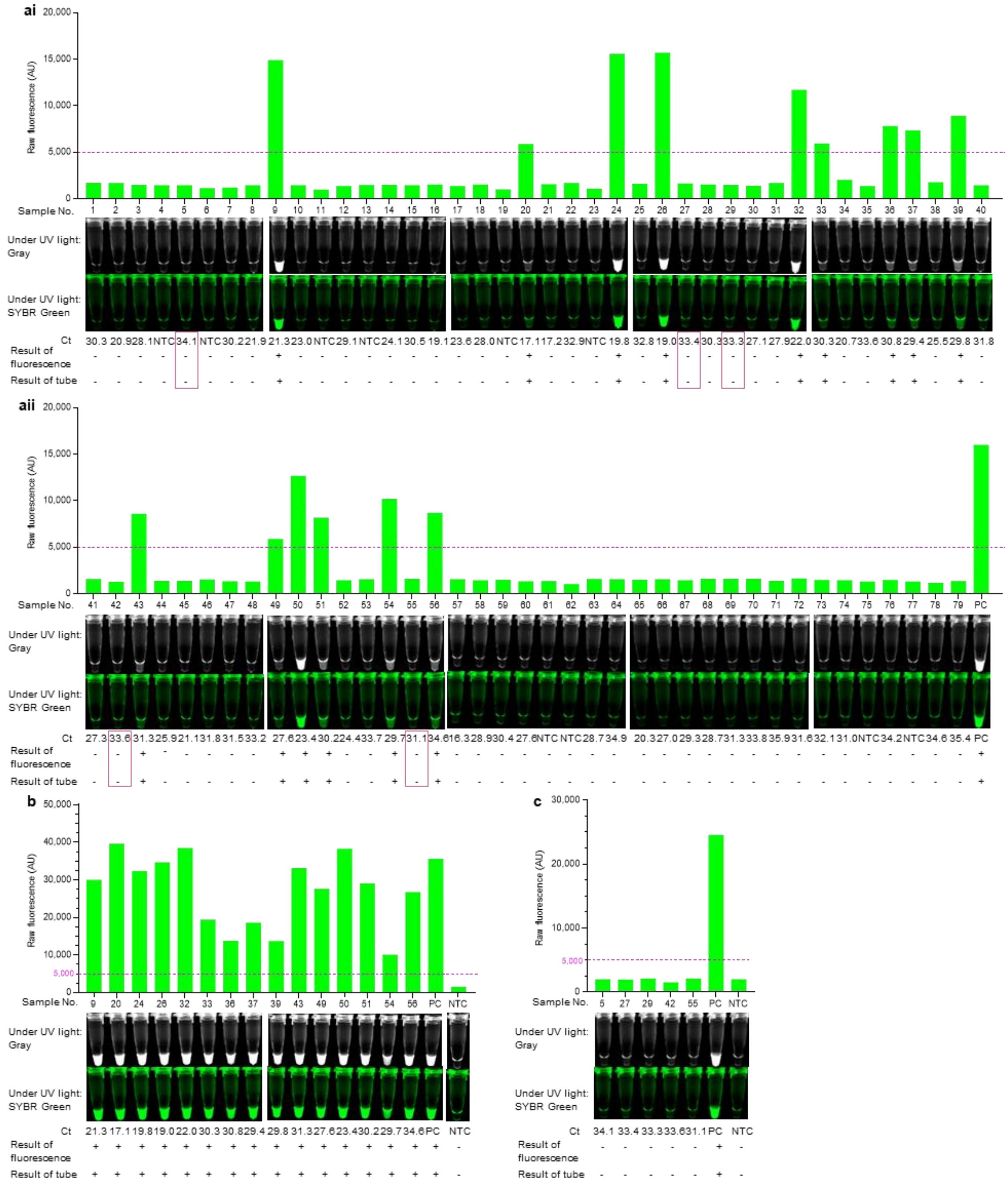


**Figure S23. Fluorescence and tube results after 30 min incubation of SAVED CRISPR-Cas12a based detection of SARS-CoV-2 Delta variants in blinded 79 samples, including 69 clinical samples and 10 NTC. ai, aii**, T478K detection (Marker of Delta; Omicron variant is not positive due to the mismatch(s) present in the T478K RPA R primer) in 79 samples, 15 in 20 were positive (Ct range: 17.1-34.6), 5 in 20 were false negative (Ct range: 31.1-34.1), no false positive (100% specificity, Ct range: 16.3-35.9), the sensitivity was 75% (Ct range: 17.1-34.6), for samples Ct < 31.1 (≤30.8) showed 100% sensitivity. **b**, L452R detection (Delta marker for double confirmation) in 15 T478K positive samples, all of them are positive (Ct range: 17.1-

34.6), with no false positive. **c**, L452R detection (Delta marker for double confirmation) in 5 T478K false-negative samples, all of them are false negative (Ct range: 31.1-34.1), no false negative. So the L452R detection shared the same sensitivity and specificity. AU means arbitrary units. The horizontal pink dash line indicates the fluorescence threshold (5000 AU) of positive samples, which can be visualized under UV light. False-negative samples are highlighted by pink rectangles.

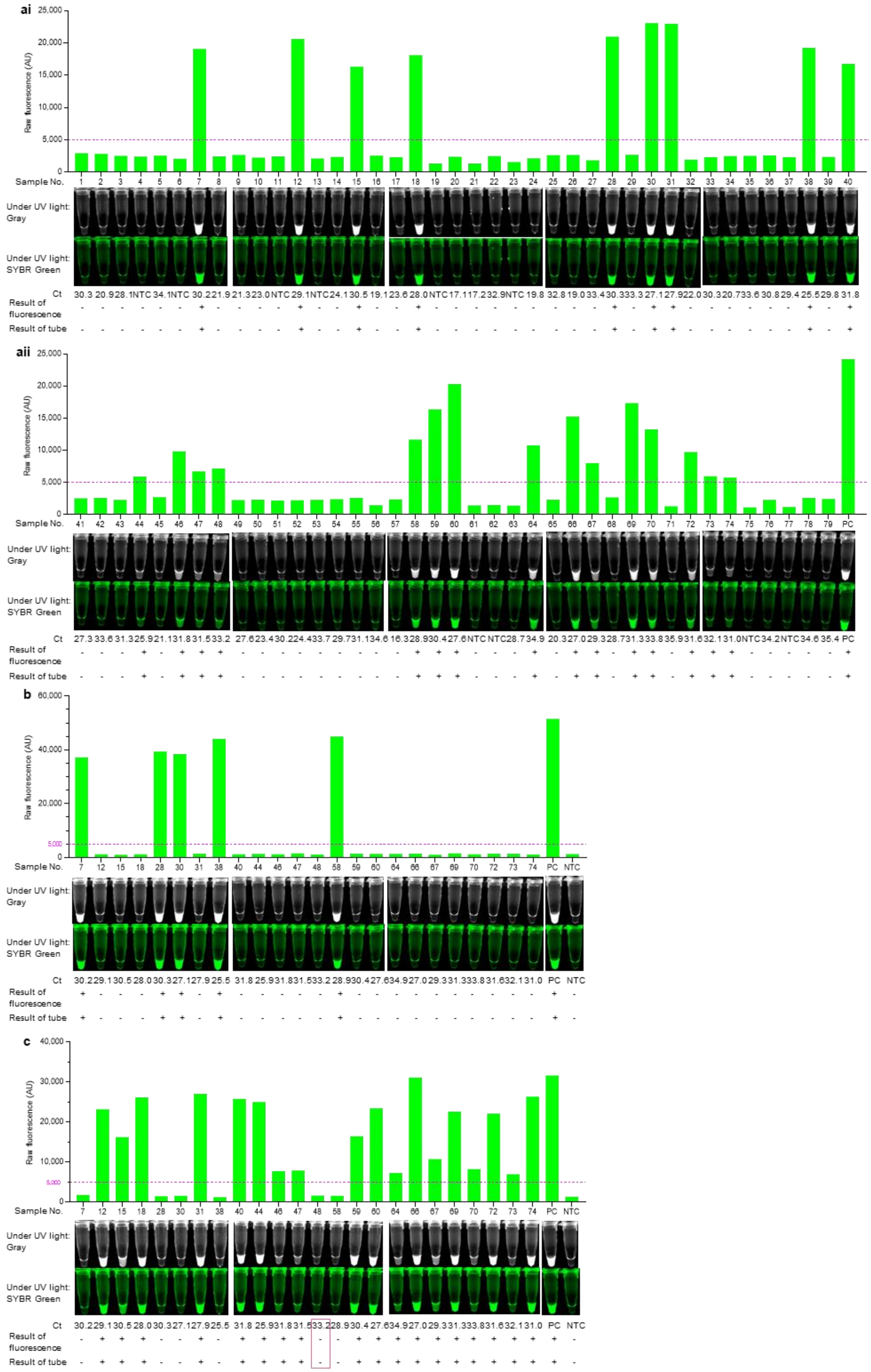
ai
aii
b
c
Raw fluorescence (AU)
Sample No.
Under UV light: Gray
Under UV light: SYBR Green
Ct
Result of fluorescence
Result of tube

**Figure S24. Fluorescence and tube results after 30 min incubation of SAVED CRISPR-Cas12a based detection of SARS-CoV-2 Omicron variant in blinded 79 samples, including 69 clinical samples and 10 NTC. ai, aii**, E484A detection (Omicron marker) in 79 samples, 24 out of 24 were positive (Ct range: 25.5-34.9, 100% sensitivity), no false positive (100% specificity, Ct range: 16.3-35.9). **b**, G142D+Δ143-145 detection (marker of BA.1, BA.1.1, and BA.3) in 24 E484A positive samples, 5 of 5 were positive (Ct range: 25.5-30.3, 100% sensitivity), no false positive (100% specificity, Ct range: 25.9-34.9). **c**, G142D detection (marker of BA.2, BA.2.12.1, BA.4, and BA.5) in 24 E484A positive samples, 18 out of 24 were positive (Ct range: 25.9-34.9), 1 was false negative (Ct: 33.2), no false positive (100% specificity, Ct range: 25.5-30.3), so the sensitivity was 94.7% (Ct range: 25.9-34.9), , for samples Ct < 33.2 (≤ 32.1) showed 100% sensitivity. AU means arbitrary units. The horizontal pink dash line indicates the fluorescence threshold (5000 AU) of positive samples, which can be visualized under UV light. The false-negative sample is highlighted by a pink rectangle.

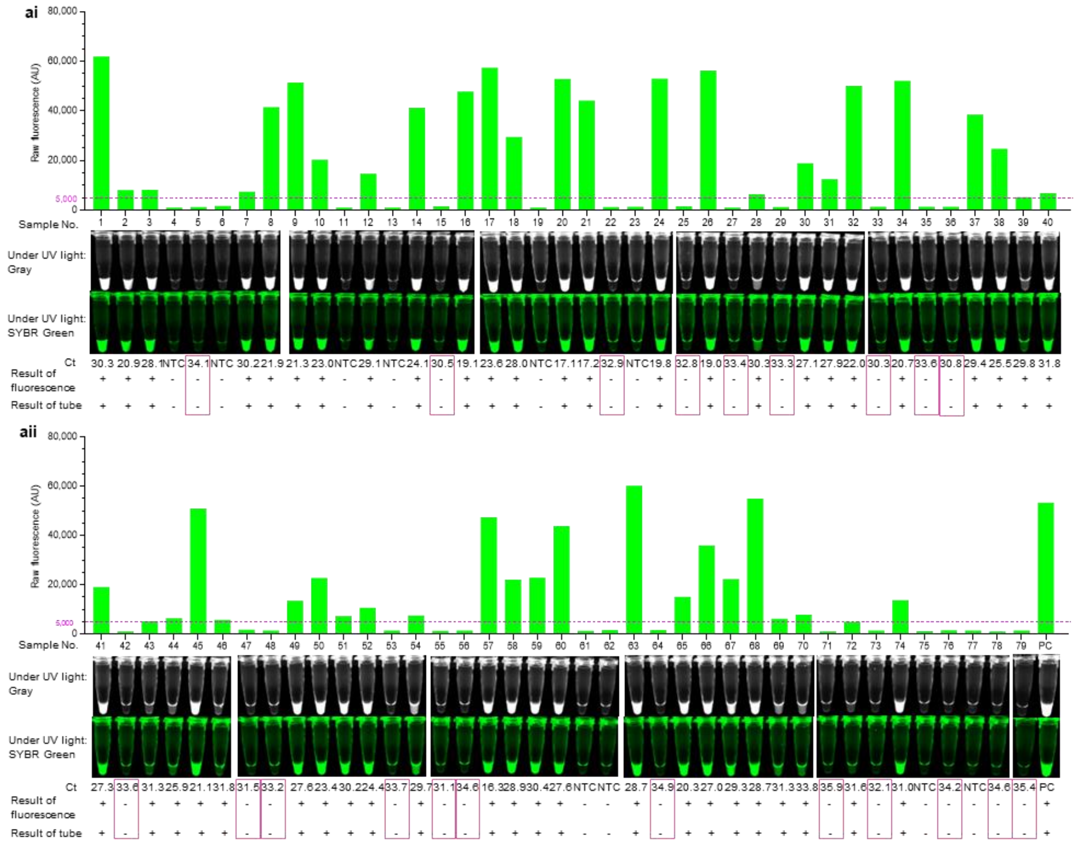


**Figure S25. Fluorescence and tube results after 30 min incubation of SAVED CRISPR-Cas12a based detection of SARS-CoV-2 (N2 region) in blinded 79 samples, including 69 clinical samples and 10 NTC. ai, aii**, SARS-CoV-2 detection (N2 region of N gene) in 79 samples, 48 in 69 were positive (Ct range: 16.3-33.8), 21 out of 69 were false negative (Ct range: 30.3-35.9), so 100% specificity as there was no false positive, the sensitivity was 69.6% (Ct: 16.3-35.9), however, for samples Ct ≤ 30.2 with 100% sensitivity. False-negative samples are highlighted by pink rectangles. AU means arbitrary units. The horizontal pink dash line indicates the fluorescence threshold (5000 AU) of positive samples, which can be visualized under UV light.

# Supplementary tables

**Table S1 crRNAs and IVT templates used in this study.**

| Name | Sequence | Target variants | REF |
|---|---|---|---|
| **Selected crRNAs and IVT templates** | | | |
| T7-3G IVT primer | GAAATTAATACGACTCACTATAGGG | NA | (1) |
| T95I crRNA 17-nt | UAAUUUCUACUAAGUGUAGAU-CUUCCAUUGAGAAGUCU | Mu, Iota | This study |
| T95I crRNA 17-nt IVT template | AGACTTCTCAATGGAAG-ATCTACACTTAGTAGAAATTA-CCCTATAGTGAGTCGTATTAATTTC | | This study |
| D138Y crRNA (12th) 15-nt | UAAUUUCUACUAAGUGUAGAU-AAUUUUGUAAUUAUC | Gamma | This study |
| D138Y crRNA (12th) 15-nt IVT template | GATAATTACAAAATT-ATCTACACTTAGTAGAAATTA-CCCTATAGTGAGTCGTATTAATTTC | | This study |
| G142D crRNA (13th) 15-nt | UAAUUUCUACUAAGUGUAGAU-UGGUAAUAAACAUCC | Omicron | This study |
| G142D crRNA (13th) 15-nt IVT template | GGATGTTTATTACCA-ATCTACACTTAGTAGAAATTA-CCCTATAGTGAGTCGTATTAATTTC | | This study |
| G142D+Δ143-145 crRNA 18-nt | UAAUUUCUACUAAGUGUAGAU-UGGUCCAAAAAUGGAUCA | Omicron | This study |
| G142D+Δ143-145 crRNA 18-nt IVT template | TGATCCATTTTTGGACCA-ATCTACACTTAGTAGAAATTA-CCCTATAGTGAGTCGTATTAATTTC | | This study |
| Δ144 crRNA 18-nt | UAAUUUCUACUAAGUGUAGAU-GGUGUUUACCACAAAAAC | Alpha, Eta | This study |
| Δ144 crRNA 18-nt IVT template | GTTTTTGTGGTAAACACC-ATCTACACTTAGTAGAAATTA-CCCTATAGTGAGTCGTATTAATTTC | | This study |
| YY144–145TSN crRNA 20-nt | UAAUUUCUACUAAGUGUAGAU-GGUGUUACUUCUAACCACAA | Mu | This study |
| YY144–145TSN crRNA 20-nt IVT template | TTGTGGTTAGAAGTAACACC-ATCTACACTTAGTAGAAATTA-CCCTATAGTGAGTCGTATTAATTTC | | This study |
| R190S crRNA 17-nt | UAAUUUCUACUAAGUGUAGAU-AAAAAUCUUAGUGAAUU | Gamma | This study |
| R190S crRNA 17-nt IVT template | AATTCACTAAGATTTTT-ATCTACACTTAGTAGAAATTA-CCCTATAGTGAGTCGTATTAATTTC | | This study |
| Δ242-244 chimeric crRNA 24-nt | UAAUUUCUACUAAGUGUAGAU-AAACUUUACAUAGAAGTTATTTGA | Beta | This study |
| R246N+Δ247-253 crRNA 20-nt | UAAUUUCUACUAAGUGUAGAU-CAUAAUUCUUCUUCAGGUUG | Lambda | This study |
| R246N+Δ247-253 crRNA 20-nt IVT | CAACCTGAAGAAGAATTATG-ATCTACACTTAGTAGAAATTA- | | This study |

| | | | |
|---|---|---|---|
| template | CCCTATAGTGAGTCGTATTAATTTC | | |
| R346K crRNA 16-nt | UAAUUUCUACUAAGUGUAGAU-ACGCCACCAAAUUUGC | Mu, Omicron BA.1.1 | This study |
| R346K crRNA 16-nt IVT template | GCAAATTTGGTGGCGT-ATCTACACTTAGTAGAAATTA-CCCTATAGTGAGTCGTATTAATTTC | | This study |
| K417N chimeric crRNA (7th) 24-nt | UAAUUUCUACUAAGUGUAGAU-UGGAAAUAUUGCUGAUTATAATTA | Beta, Omicron | This study |
| K417T crRNA (6th) 16-nt | UAAUUUCUACUAAGUGUAGAU-UGGAACGAUUGCUGAU | Gamma | This study |
| K417T crRNA (6th) 16-nt template | ATCAGCAATCGTTCCA-ATCTACACTTAGTAGAAATTA-CCCTATAGTGAGTCGTATTAATTTC | | This study |
| L452Q crRNA (11th) 16-nt | UAAUUUCUACUAAGUGUAGAU-UAUAAUUACCAGUAUA | Lambda, Omicron BA.2.12.1 | This study |
| L452Q crRNA (11th) 16-nt IVT template | TATACTGGTAATTATA-ATCTACACTTAGTAGAAATTA-CCCTATAGTGAGTCGTATTAATTTC | | This study |
| L452R crRNA (11th) 16-nt | UAAUUUCUACUAAGUGUAGAU-UAUAAUUACCGGUAUA | Delta, Kappa, Epsilon, Omicron BA.4, BA.5 | This study |
| L452R crRNA (11th) 16-nt IVT template | TATACCGGTAATTATA-ATCTACACTTAGTAGAAATTA-CCCTATAGTGAGTCGTATTAATTTC | | This study |
| T478K chimeric crRNA 24-nt | UAAUUUCUACUAAGUGUAGAU-CUACCGGCCUGAUAGATTTCAGTT | Delta, Omicron | This study |
| E484K crRNA (11th) 15-nt | UAAUUUCUACUAAGUGUAGAU-UAAUGGUGUUAAAGG | Beta, Gamma, Mu, Zeta, Eta, Theta | This study |
| E484K crRNA (11th) 15-nt IVT template | CCTTTAACACCATTA-ATCTACACTTAGTAGAAATTA-CCCTATAGTGAGTCGTATTAATTTC | | This study |
| E484Q crRNA (11th) 15-nt | UAAUUUCUACUAAGUGUAGAU-UAAUGGUGUUCAAGG | Kappa | This study |
| E484Q crRNA (11th) 15-nt IVT template | CCTTGAACACCATTA-ATCTACACTTAGTAGAAATTA-CCCTATAGTGAGTCGTATTAATTTC | | This study |
| E484A crRNA (12th) 15-nt | UAAUUUCUACUAAGUGUAGAU-UAAUGGUGUUGCAGG | Omicron | This study |
| E484A crRNA (12th) 15-nt IVT template | CCTGCAACACCATTA-ATCTACACTTAGTAGAAATTA-CCCTATAGTGAGTCGTATTAATTTC | | This study |
| F490S crRNA 17-nt | UAAUUUCUACUAAGUGUAGAU-AUUGUUACUCUCCUUUA | Lambda | This study |
| F490S crRNA 17-nt IVT template | TAAAGGAGAGTAACAAT-ATCTACACTTAGTAGAAATTA-CCCTATAGTGAGTCGTATTAATTTC | | This study |

| | | | |
|---|---|---|---|
| G493R+G496S crRNA 16-nt | UAAUUUCUACUAAGUGUAGAU-CGAUCAUAUAGUUUCC | Omicron BA.1, BA.1.1 | This study |
| G493R+G496S crRNA 16-nt IVT template | GGAAACTATATGATCG-ATCTACACTTAGTAGAAATTA-CCCTATAGTGAGTCGTATTAATTTC | | This study |
| N501Y chimeric crRNA 24-nt | UAAUUUCUACUAAGUGUAGAU-CAACCCACUUAUGGUGTTGGTTAC | Alpha, Beta, Gamma, Omicron, Mu, Theta | (2) |
| N2 crRNA 20-nt | UAAUUUCUACUAAGUGUAGAU-CCCCCAGCGCUUCAGCGUUC | SARS-CoV-2 | (3) |
| N2 crRNA 20-nt IVT template | GAACGCTGAAGCGCTGGGGG-ATCTACACTTAGTAGAAATTA-CCCTATAGTGAGTCGTATTAATTTC | | (3) |
| **Additional crRNAs and IVT templates** | | | |
| T95I crRNA 18-nt | UAAUUUCUACUAAGUGUAGAU-CUUCCAUUGAGAAGUCUA | Rejected | This study |
| T95I crRNA 18-nt IVT template | TAGACTTCTCAATGGAAG-ATCTACACTTAGTAGAAATTA-CCCTATAGTGAGTCGTATTAATTTC | Rejected | This study |
| T95I crRNA 16-nt | UAAUUUCUACUAAGUGUAGAU-CUUCCAUUGAGAAGUC | Rejected | This study |
| T95I crRNA 16-nt IVT template | GACTTCTCAATGGAAG-ATCTACACTTAGTAGAAATTA-CCCTATAGTGAGTCGTATTAATTTC | Rejected | This study |
| D138Y crRNA (5th) 16-nt | UAAUUUCUACUAAGUGUAGAU-UAAUUAUCCAUUUUUG | Rejected | This study |
| D138Y crRNA (5th) 16-nt IVT template | CAAAAATGGATAATTA-ATCTACACTTAGTAGAAATTA-CCCTATAGTGAGTCGTATTAATTTC | Rejected | This study |
| D138Y crRNA (12th) 16-nt | UAAUUUCUACUAAGUGUAGAU-AAUUUUGUAAUUAUCC | Rejected | This study |
| D138Y crRNA (12th) 16-nt IVT template | GGATAATTACAAAATT-ATCTACACTTAGTAGAAATTA-CCCTATAGTGAGTCGTATTAATTTC | Rejected | This study |
| G142D crRNA (4th) 15-nt | UAAUUUCUACUAAGUGUAGAU-UGGAUGUUUAUUACC | Rejected | This study |
| G142D crRNA (4th) 15-nt IVT template | GGTAATAAACATCCA-ATCTACACTTAGTAGAAATTA-CCCTATAGTGAGTCGTATTAATTTC | Rejected | This study |
| G142D crRNA (4th) 16-nt | UAAUUUCUACUAAGUGUAGAU-UGGAUGUUUAUUACCU | Rejected | This study |
| G142D crRNA (4th) 16-nt IVT | TGGTAATAAACATCCA-ATCTACACTTAGTAGAAATTA- | Rejected | This study |

| | | | |
|---|---|---|---|
| template | CCCTATAGTGAGTCGTATTAATTTC | | |
| G142D+Δ143-145 crRNA 20-nt | UAAUUUCUACUAAGUGUAGAU-UGGUCCAAAAAUGGAUCAUU | Rejected | This study |
| G142D+Δ143-145 crRNA 20-nt IVT template | AATGATCCATTTTTGGACCA-ATCTACACTTAGTAGAAATTA-CCCTATAGTGAGTCGTATTAATTTC | Rejected | This study |
| Δ144 crRNA 20-nt | UAAUUUCUACUAAGUGUAGAU-GGUGUUUACCACAAAAACAA | Rejected | This study |
| Δ144 crRNA 20-nt IVT template | TTGTTTTTGTGGTAAACACC-ATCTACACTTAGTAGAAATTA-CCCTATAGTGAGTCGTATTAATTTC | Rejected | This study |
| Δ144 crRNA 17-nt | UAAUUUCUACUAAGUGUAGAU-GGUGUUUACCACAAAAA | Rejected | This study |
| Δ144 crRNA 17-nt IVT template | TTTTTGTGGTAAACACC-ATCTACACTTAGTAGAAATTA-CCCTATAGTGAGTCGTATTAATTTC | Rejected | This study |
| Δ144 crRNA 16-nt | UAAUUUCUACUAAGUGUAGAU-GGUGUUUACCACAAAA | Rejected | This study |
| Δ144 crRNA 16-nt IVT template | TTTTGTGGTAAACACC-ATCTACACTTAGTAGAAATTA-CCCTATAGTGAGTCGTATTAATTTC | Rejected | This study |
| YY144–145TSN crRNA 16-nt | UAAUUUCUACUAAGUGUAGAU-GGUGUUACUUCUAACC | Rejected | This study |
| YY144–145TSN crRNA 16-nt IVT template | GGTTAGAAGTAACACC-ATCTACACTTAGTAGAAATTA-CCCTATAGTGAGTCGTATTAATTTC | Rejected | This study |
| R190S crRNA 18-nt | UAAUUUCUACUAAGUGUAGAU-AAAAAUCUUAGUGAAUUU | Rejected | This study |
| R190S crRNA 18-nt IVT template | AAATTCACTAAGATTTTT-ATCTACACTTAGTAGAAATTA-CCCTATAGTGAGTCGTATTAATTTC | Rejected | This study |
| R190S crRNA 16-nt | UAAUUUCUACUAAGUGUAGAU-AAAAAUCUUAGUGAAU | Rejected | This study |
| R190S crRNA 16-nt IVT template | ATTCACTAAGATTTTT-ATCTACACTTAGTAGAAATTA-CCCTATAGTGAGTCGTATTAATTTC | Rejected | This study |
| Δ242-244 crRNA 24-nt | UAAUUUCUACUAAGUGUAGAU-AAACUUUACAUAGAAGUUAUUUGA | Rejected | This study |
| Δ242-244 crRNA 24-nt IVT template | TCAAATAACTTCTATGTAAAGTTT-ATCTACACTTAGTAGAAATTA-CCCTATAGTGAGTCGTATTAATTTC | Rejected | This study |

| | | | |
|---|---|---|---|
| Δ242-244 crRNA 20-nt | UAAUUUCUACUAAGUGUAGAU-AAACUUUACAUAGAAGUUAU | Rejected | This study |
| Δ242-244 crRNA 20-nt IVT template | ATAACTTCTATGTAAAGTTT-ATCTACACTTAGTAGAAATTA-CCCTATAGTGAGTCGTATTAATTTC | Rejected | This study |
| R346K crRNA 18-nt | UAAUUUCUACUAAGUGUAGAU-ACGCCACCAAAUUUGCAU | Rejected | This study |
| R346K crRNA 18-nt IVT template | ATGCAAATTTGGTGGCGT-ATCTACACTTAGTAGAAATTA-CCCTATAGTGAGTCGTATTAATTTC | Rejected | This study |
| R346K crRNA 17-nt | UAAUUUCUACUAAGUGUAGAU-ACGCCACCAAAUUUGCA | Rejected | This study |
| R346K crRNA 17-nt IVT template | TGCAAATTTGGTGGCGT-ATCTACACTTAGTAGAAATTA-CCCTATAGTGAGTCGTATTAATTTC | Rejected | This study |
| R346K crRNA 15-nt | UAAUUUCUACUAAGUGUAGAU-ACGCCACCAAAUUUG | Rejected | This study |
| R346K crRNA 15-nt IVT template | CAAATTTGGTGGCGT-ATCTACACTTAGTAGAAATTA-CCCTATAGTGAGTCGTATTAATTTC | Rejected | This study |
| K417N crRNA (7th) 24-nt | UAAUUUCUACUAAGUGUAGAU-UGGAAAUAUUGCUGAUUAUAAUUA | Rejected | This study |
| K417N crRNA (7th) 24-nt IVT template | TAATTATAATCAGCAATATTTCCA-ATCTACACTTAGTAGAAATTA-CCCTATAGTGAGTCGTATTAATTTC | Rejected | This study |
| K417N crRNA (7th) 20-nt | UAAUUUCUACUAAGUGUAGAU-UGGAAAUAUUGCUGAUUAUA | Rejected | This study |
| K417N crRNA (7th) 20-nt IVT template | TATAATCAGCAATATTTCCA-ATCTACACTTAGTAGAAATTA-CCCTATAGTGAGTCGTATTAATTTC | Rejected | This study |
| K417N crRNA (7th) 17-nt | UAAUUUCUACUAAGUGUAGAU-UGGAAAUAUUGCUGAUU | Rejected | This study |
| K417N crRNA (7th) 17-nt IVT template | AATCAGCAATATTTCCA-ATCTACACTTAGTAGAAATTA-CCCTATAGTGAGTCGTATTAATTTC | Rejected | This study |
| K417N crRNA (7th) 16-nt | UAAUUUCUACUAAGUGUAGAU-UGGAAAUAUUGCUGAU | Rejected | This study |
| K417N crRNA (7th) 16-nt IVT template | ATCAGCAATATTTCCA-ATCTACACTTAGTAGAAATTA-CCCTATAGTGAGTCGTATTAATTTC | Rejected | This study |
| K417N crRNA (11th) 16-nt | UAAUUUCUACUAAGUGUAGAU-AAACUGGAAAUAUUGC | Rejected | This study |

| | | | |
|---|---|---|---|
| K417N crRNA (11th) 16-nt IVT template | GCAATATTTCCAGTTT-ATCTACACTTAGTAGAAATTA-CCCTATAGTGAGTCGTATTAATTTC | Rejected | This study |
| K417T chimeric crRNA (19th) 24-nt | UAAUUUCUACUAAGUGUAGAU-UAAUUAUAAUCAGCAATCGTTCCA | Rejected | This study |
| K417T chimeric crRNA (6th) 24-nt | UAAUUUCUACUAAGUGUAGAU-UGGAACGAUUGCUGAUTATAATTA | Rejected | This study |
| K417T crRNA (6th) 24-nt | UAAUUUCUACUAAGUGUAGAU-UGGAACGAUUGCUGAUUAUAAUUA | Rejected | This study |
| K417T crRNA (6th) 24-nt IVT template | TAATTATAATCAGCAATCGTTCCA- ATCTACACTTAGTAGAAATTA-CCCTATAGTGAGTCGTATTAATTTC | Rejected | This study |
| K417T crRNA (6th) 20-nt | UAAUUUCUACUAAGUGUAGAU-UGGAACGAUUGCUGAUUAUA | Rejected | This study |
| K417T crRNA (6th) 20-nt IVT template | TATAATCAGCAATCGTTCCA-ATCTACACTTAGTAGAAATTA-CCCTATAGTGAGTCGTATTAATTTC | Rejected | This study |
| K417T crRNA (6th) 17-nt | UAAUUUCUACUAAGUGUAGAU-UGGAACGAUUGCUGAUU | Rejected | This study |
| K417T crRNA (6th) 17-nt IVT template | AATCAGCAATCGTTCCA-ATCTACACTTAGTAGAAATTA-CCCTATAGTGAGTCGTATTAATTTC | Rejected | This study |
| K417T chimeric crRNA (10th) 24-nt | UAAUUUCUACUAAGUGUAGAU-AAACUGGAACGAUUGCTGATTATA | Rejected | This study |
| K417T crRNA (10th9U) 24-nt | UAAUUUCUACUAAGUGUAGAU-AAACUGGAUCGAUUGCUGAUUAUA | Rejected | This study |
| K417T crRNA (10th9U) 24-nt IVT template | TATAATCAGCAATCGATCCAGTTT-ATCTACACTTAGTAGAAATTA-CCCTATAGTGAGTCGTATTAATTTC | Rejected | This study |
| K417T crRNA (10th9C) 24-nt | UAAUUUCUACUAAGUGUAGAU-AAACUGGACCGAUUGCUGAUUAUA | Rejected | This study |
| K417T crRNA (10th9C) 24-nt IVT template | TATAATCAGCAATCGGTCCAGTTT-ATCTACACTTAGTAGAAATTA-CCCTATAGTGAGTCGTATTAATTTC | Rejected | This study |
| K417T crRNA (10th8G9C) 24-nt | UAAUUUCUACUAAGUGUAGAU-AAACUGGGCCGAUUGCUGAUUAUA | Rejected | This study |
| K417T crRNA (10th8G9C) 24-nt | TATAATCAGCAATCGGCCCAGTTT-ATCTACACTTAGTAGAAATTA- | Rejected | This study |

| | | | |
|---|---|---|---|
| IVT template | CCCTATAGTGAGTCGTATTAATTTC | | |
| K417T chimeric crRNA (10th) 3' DNA7 27-nt | UAAUUUCUACUAAGUGUAGAU-AAACUGGAACGAUUGCUGAUTATTATT | Rejected | This study |
| K417T crRNA (10th) 17-nt | UAAUUUCUACUAAGUGUAGAU-AAACUGGAACGAUUGCU | Rejected | This study |
| K417T crRNA (10th) 17-nt IVT template | AGCAATCGTTCCAGTTT-ATCTACACTTAGTAGAAATTA-CCCTATAGTGAGTCGTATTAATTTC | Rejected | This study |
| K417T crRNA (10th) 16-nt | UAAUUUCUACUAAGUGUAGAU-AAACUGGAACGAUUGC | Rejected | This study |
| K417T crRNA (10th) 16-nt IVT template | GCAATCGTTCCAGTTT-ATCTACACTTAGTAGAAATTA-CCCTATAGTGAGTCGTATTAATTTC | Rejected | This study |
| K417T crRNA (10th9G) 16-nt | UAAUUUCUACUAAGUGUAGAU-AAACUGGAGCGAUUGC | Rejected | This study |
| K417T crRNA (10th9G) 16-nt IVT template | GCAATCGCTCCAGTTT-ATCTACACTTAGTAGAAATTA-CCCTATAGTGAGTCGTATTAATTTC | Rejected | This study |
| L452Q crRNA (7th) 16-nt | UAAUUUCUACUAAGUGUAGAU-AUUACCAGUAUAGAUU | Rejected | This study |
| L452Q crRNA (7th) 16-nt IVT template | AATCTATACTGGTAAT-ATCTACACTTAGTAGAAATTA-CCCTATAGTGAGTCGTATTAATTTC | Rejected | This study |
| L452Q crRNA (7th) 15-nt | UAAUUUCUACUAAGUGUAGAU-AUUACCAGUAUAGAU | Rejected | This study |
| L452Q crRNA (7th) 15-nt IVT template | ATCTATACTGGTAAT-ATCTACACTTAGTAGAAATTA-CCCTATAGTGAGTCGTATTAATTTC | Rejected | This study |
| L452Q chimeric crRNA (9th) 24-nt | UAAUUUCUACUAAGUGUAGAU-UAAUUACCAGUAUAGATTGTTTAG | Rejected | This study |
| L452Q chimeric crRNA (9th) 3' DNA7 23-nt | UAAUUUCUACUAAGUGUAGAU-UAAUUACCAGUAUAGATATTATT | Rejected | This study |
| L452Q crRNA (9th) 16-nt | UAAUUUCUACUAAGUGUAGAU-UAAUUACCAGUAUAGA | Rejected | This study |
| L452Q crRNA (9th) 16-nt IVT template | TCTATACTGGTAATTA-ATCTACACTTAGTAGAAATTA-CCCTATAGTGAGTCGTATTAATTTC | Rejected | This study |
| L452Q crRNA (9th8U) 16-nt | UAAUUUCUACUAAGUGUAGAU-UAAUUACUAGUAUAGA | Rejected | This study |
| L452Q crRNA (9th8U) 16-nt IVT template | TCTATACTAGTAATTA-ATCTACACTTAGTAGAAATTA-CCCTATAGTGAGTCGTATTAATTTC | Rejected | This study |
| L452Q crRNA (9th) 15-nt | UAAUUUCUACUAAGUGUAGAU-UAAUUACCAGUAUAG | Rejected | This study |

| | | | |
|---|---|---|---|
| L452Q crRNA (9th) 15-nt IVT template | CTATACTGGTAATTA-ATCTACACTTAGTAGAAATTA-CCCTATAGTGAGTCGTATTAATTTC | Rejected | This study |
| L452Q crRNA (10th) 16-nt | UAAUUUCUACUAAGUGUAGAU-AUAAUUACCAGUAUAG | Rejected | This study |
| L452Q crRNA (10th) 16-nt IVT template | CTATACTGGTAATTAT-ATCTACACTTAGTAGAAATTA-CCCTATAGTGAGTCGTATTAATTTC | Rejected | This study |
| L452Q crRNA (10th) 15-nt | UAAUUUCUACUAAGUGUAGAU-AUAAUUACCAGUAUA | Rejected | This study |
| L452Q crRNA (10th) 15-nt IVT template | TATACTGGTAATTAT-ATCTACACTTAGTAGAAATTA-CCCTATAGTGAGTCGTATTAATTTC | Rejected | This study |
| L452Q chimeric crRNA (11th) 3' DNA7 22-nt | UAAUUUCUACUAAGUGUAGAU-UAUAAUUACCAGUAUTATTATT | Rejected | This study |
| L452Q crRNA (11th) 15-nt | UAAUUUCUACUAAGUGUAGAU-UAUAAUUACCAGUAU | Rejected | This study |
| L452Q crRNA (11th) 15-nt IVT template | ATACTGGTAATTATA-ATCTACACTTAGTAGAAATTA-CCCTATAGTGAGTCGTATTAATTTC | Rejected | This study |
| L452Q crRNA (12th) 16-nt | UAAUUUCUACUAAGUGUAGAU-UUAUAAUUACCAGUAU | Rejected | This study |
| L452Q crRNA (12th) 16-nt IVT template | ATACTGGTAATTATAA-ATCTACACTTAGTAGAAATTA-CCCTATAGTGAGTCGTATTAATTTC | Rejected | This study |
| L452Q crRNA (12th) 15-nt | UAAUUUCUACUAAGUGUAGAU-UUAUAAUUACCAGUA | Rejected | This study |
| L452Q crRNA (12th) 15-nt IVT template | TACTGGTAATTATAA-ATCTACACTTAGTAGAAATTA-CCCTATAGTGAGTCGTATTAATTTC | Rejected | This study |
| L452R chimeric crRNA (7th) 24-nt | UAAUUUCUACUAAGUGUAGAU-AUUACCGGUAUAGAUUGTTTAGGA | Rejected | This study |
| L452R crRNA (7th) 24-nt | UAAUUUCUACUAAGUGUAGAU-AUUACCGGUAUAGAUUGUUUAGGA | Rejected | This study |
| L452R crRNA (7th) 24-nt IVT template | TCCTAAACAATCTATACCGGTAAT-ATCTACACTTAGTAGAAATTA-CCCTATAGTGAGTCGTATTAATTTC | Rejected | This study |
| L452R crRNA (7th) 20-nt | UAAUUUCUACUAAGUGUAGAU-AUUACCGGUAUAGAUUGUUU | Rejected | This study |
| L452R crRNA (7th) 20-nt IVT template | AAACAATCTATACCGGTAAT-ATCTACACTTAGTAGAAATTA-CCCTATAGTGAGTCGTATTAATTTC | Rejected | This study |
| L452R chimeric crRNA (7th6G) 24-nt | UAAUUUCUACUAAGUGUAGAU-AUUACGGGUAUAGAUUGTTTAGGA | Rejected | This study |
| L452R crRNA | UAAUUUCUACUAAGUGUAGAU- | Rejected | This |

| | | | |
|---|---|---|---|
| (7th6G) 24-nt | AUUACGGGUAUAGAUUGUUAGGA | | study |
| L452R crRNA (7th6G) 24-nt IVT template | TCCTAAACAATCTATACCCGTAAT-ATCTACACTTAGTAGAAATTA-CCCTATAGTGAGTCGTATTAATTTC | Rejected | This study |
| L452R chimeric crRNA (7th5G) 24-nt | UAAUUUCUACUAAGUGUAGAU-AUUAGCGGUAUAGAUUGTTTAGGA | Rejected | This study |
| L452R chimeric crRNA (7th) 3' DNA7 27-nt | UAAUUUCUACUAAGUGUAGAU-AUUACCGGUAUAGAUUGUUUTATTATT | Rejected | This study |
| L452R crRNA (7th) 16-nt | UAAUUUCUACUAAGUGUAGAU-AUUACCGGUAUAGAUU | Rejected | This study |
| L452R crRNA (7th) 16-nt IVT template | AATCTATACCGGTAAT-ATCTACACTTAGTAGAAATTA-CCCTATAGTGAGTCGTATTAATTTC | Rejected | This study |
| L452R crRNA (7th6U) 16-nt | UAAUUUCUACUAAGUGUAGAU-AUUACUGGUAUAGAUU | Rejected | This study |
| L452R crRNA (7th6U) 16-nt IVT template | AATCTATACCAGTAAT-ATCTACACTTAGTAGAAATTA-CCCTATAGTGAGTCGTATTAATTTC | Rejected | This study |
| L452R crRNA (7th6U8A) 16-nt | UAAUUUCUACUAAGUGUAGAU-AUUACUGAUAUAGAUU | Rejected | This study |
| L452R crRNA (7th6U8A) 16-nt IVT template | AATCTATATCAGTAAT-ATCTACACTTAGTAGAAATTA-CCCTATAGTGAGTCGTATTAATTTC | Rejected | This study |
| L452R crRNA (7th) 15-nt | UAAUUUCUACUAAGUGUAGAU-AUUACCGGUAUAGAU | Rejected | This study |
| L452R crRNA (7th) 15-nt IVT template | ATCTATACCGGTAAT-ATCTACACTTAGTAGAAATTA-CCCTATAGTGAGTCGTATTAATTTC | Rejected | This study |
| L452R chimeric crRNA (9th) 24-nt | UAAUUUCUACUAAGUGUAGAU-UAAUUACCGGUAUAGATTGTTTAG | Rejected | This study |
| L452R chimeric crRNA (9th) 3' DNA7 27-nt | UAAUUUCUACUAAGUGUAGAU-UAAUUACCGGUAUAGAUUGUTATTATT | Rejected | This study |
| L452R chimeric crRNA (9th) 3' DNA7 23-nt | UAAUUUCUACUAAGUGUAGAU-UAAUUACCGGUAUAGATATTATT | Rejected | This study |
| L452R crRNA (9th) 16-nt | UAAUUUCUACUAAGUGUAGAU-UAAUUACCGGUAUAGA | Rejected | This study |
| L452R crRNA (9th) 16-nt IVT template | TCTATACCGGTAATTA-ATCTACACTTAGTAGAAATTA- | Rejected | This study |

| | | | |
|---|---|---|---|
| | CCCTATAGTGAGTCGTATTAATTTC | | |
| L452R crRNA (9th8U) 16-nt | UAAUUUCUACUAAGUGUAGAU-UAAUUACUGGUAUAGA | Rejected | This study |
| L452R crRNA (9th8U) 16-nt IVT template | TCTATACCAGTAATTA-ATCTACACTTAGTAGAAATTA-CCCTATAGTGAGTCGTATTAATTTC | Rejected | This study |
| L452R crRNA (9th8A) 16-nt | UAAUUUCUACUAAGUGUAGAU-UAAUUACAGGUAUAGA | Rejected | This study |
| L452R crRNA (9th8A) 16-nt IVT template | TCTATACCTGTAATTA-ATCTACACTTAGTAGAAATTA-CCCTATAGTGAGTCGTATTAATTTC | Rejected | This study |
| L452R crRNA (9th8G) 16-nt | UAAUUUCUACUAAGUGUAGAU-UAAUUACGGGUAUAGA | Rejected | This study |
| L452R crRNA (9th8G) 16-nt IVT template | TCTATACCCGTAATTA-ATCTACACTTAGTAGAAATTA-CCCTATAGTGAGTCGTATTAATTTC | Rejected | This study |
| L452R crRNA (9th10A) 16-nt | UAAUUUCUACUAAGUGUAGAU-UAAUUACCGAUAUAGA | Rejected | This study |
| L452R crRNA (9th10A) 16-nt IVT template | TCTATATCGGTAATTA-ATCTACACTTAGTAGAAATTA-CCCTATAGTGAGTCGTATTAATTTC | Rejected | This study |
| L452R crRNA (9th10C) 16-nt | UAAUUUCUACUAAGUGUAGAU-UAAUUACCGCUAUAGA | Rejected | This study |
| L452R crRNA (9th10C) 16-nt IVT template | TCTATAGCGGTAATTA-ATCTACACTTAGTAGAAATTA-CCCTATAGTGAGTCGTATTAATTTC | Rejected | This study |
| L452R crRNA (9th10U) 16-nt | UAAUUUCUACUAAGUGUAGAU-UAAUUACCGUUAUAGA | Rejected | This study |
| L452R crRNA (9th10U) 16-nt IVT template | TCTATAACGGTAATTA-ATCTACACTTAGTAGAAATTA-CCCTATAGTGAGTCGTATTAATTTC | Rejected | This study |
| L452R crRNA (9th8U10A) 16-nt | UAAUUUCUACUAAGUGUAGAU-UAAUUACUGAUAUAGA | Rejected | This study |
| L452R crRNA (9th8U10A) 16-nt IVT template | TCTATATCAGTAATTA-ATCTACACTTAGTAGAAATTA-CCCTATAGTGAGTCGTATTAATTTC | Rejected | This study |
| L452R crRNA (9th) 15-nt | UAAUUUCUACUAAGUGUAGAU-UAAUUACCGGUAUAG | Rejected | This study |
| L452R crRNA (9th) 15-nt IVT template | CTATACCGGTAATTA-ATCTACACTTAGTAGAAATTA-CCCTATAGTGAGTCGTATTAATTTC | Rejected | This study |
| L452R crRNA (10th) 16-nt | UAAUUUCUACUAAGUGUAGAU-AUAAUUACCGGUAUAG | Rejected | This study |
| L452R crRNA (10th) 16-nt IVT template | CTATACCGGTAATTAT-ATCTACACTTAGTAGAAATTA-CCCTATAGTGAGTCGTATTAATTTC | Rejected | This study |
| L452R crRNA (10th11C) 16-nt | UAAUUUCUACUAAGUGUAGAU-AUAAUUACCGCUAUAG | Rejected | This study |
| L452R crRNA | CTATAGCGGTAATTAT- | Rejected | This |

| | | | |
|---|---|---|---|
| (10th11C) 16-nt IVT template | ATCTACACTTAGTAGAAATTA-CCCTATAGTGAGTCGTATTAATTTC | | study |
| L452R crRNA (10th) 15-nt | UAAUUUCUACUAAGUGUAGAU-AUAAUUACCGGUAUA | Rejected | This study |
| L452R crRNA (10th) 15-nt IVT template | TATACCGGTAATTAT-ATCTACACTTAGTAGAAATTA-CCCTATAGTGAGTCGTATTAATTTC | Rejected | This study |
| L452R crRNA (11th9G) 16-nt | UAAUUUCUACUAAGUGUAGAU-UAUAAUUAGCGGUAUA | Rejected | This study |
| L452R crRNA (11th9G) 16-nt IVT template | TATACCGCTAATTATA-ATCTACACTTAGTAGAAATTA-CCCTATAGTGAGTCGTATTAATTTC | Rejected | This study |
| L452R chimeric crRNA (11th) 3’ DNA7 22-nt | UAAUUUCUACUAAGUGUAGAU-UAUAAUUACCGGUAUTATTATT | Rejected | This study |
| L452R crRNA (11th) 15-nt | UAAUUUCUACUAAGUGUAGAU-UAUAAUUACCGGUAU | Rejected | This study |
| L452R crRNA (11th) 15-nt IVT template | ATACCGGTAATTATA-ATCTACACTTAGTAGAAATTA-CCCTATAGTGAGTCGTATTAATTTC | Rejected | This study |
| L452R crRNA (12th) 16-nt | UAAUUUCUACUAAGUGUAGAU-UUAUAAUUACCGGUAU | Rejected | This study |
| L452R crRNA (12th) 16-nt IVT template | ATACCGGTAATTATAA-ATCTACACTTAGTAGAAATTA-CCCTATAGTGAGTCGTATTAATTTC | Rejected | This study |
| L452R crRNA (12th) 15-nt | UAAUUUCUACUAAGUGUAGAU-UUAUAAUUACCGGUA | Rejected | This study |
| L452R crRNA (12th) 15-nt IVT template | TACCGGTAATTATAA-ATCTACACTTAGTAGAAATTA-CCCTATAGTGAGTCGTATTAATTTC | Rejected | This study |
| T478K crRNA 20-nt | UAAUUUCUACUAAGUGUAGAU-CUACCGGCCUGAUAGAUUUC | Rejected | This study |
| T478K crRNA 20-nt IVT template | GAAATCTATCAGGCCGGTAG-ATCTACACTTAGTAGAAATTA-CCCTATAGTGAGTCGTATTAATTTC | Rejected | This study |
| T478K crRNA 18-nt | UAAUUUCUACUAAGUGUAGAU-CUACCGGCCUGAUAGAUU | Rejected | This study |
| T478K crRNA 18-nt IVT template | AATCTATCAGGCCGGTAG-ATCTACACTTAGTAGAAATTA-CCCTATAGTGAGTCGTATTAATTTC | Rejected | This study |
| T478K crRNA 17-nt | UAAUUUCUACUAAGUGUAGAU-CUACCGGCCUGAUAGAU | Rejected | This study |
| T478K crRNA 17-nt IVT template | ATCTATCAGGCCGGTAG-ATCTACACTTAGTAGAAATTA-CCCTATAGTGAGTCGTATTAATTTC | Rejected | This study |
| T478K crRNA 16-nt | UAAUUUCUACUAAGUGUAGAU-CUACCGGCCUGAUAGA | Rejected | This study |
| T478K crRNA 16- | TCTATCAGGCCGGTAG- | Rejected | This |

| | | | |
|---|---|---|---|
| nt IVT template | ATCTACACTTAGTAGAAATTA-CCCTATAGTGAGTCGTATTAATTTC | | study |
| E484A crRNA (10th) 15-nt | UAAUUUCUACUAAGUGUAGAU-AUGGUGUUGCAGGUU | Rejected | This study |
| E484A crRNA (10th) 15-nt IVT template | AACCTGCAACACCAT-ATCTACACTTAGTAGAAATTA-CCCTATAGTGAGTCGTATTAATTTC | Rejected | This study |
| E484A crRNA (10th) 16-nt | UAAUUUCUACUAAGUGUAGAU-AUGGUGUUGCAGGUUU | Rejected | This study |
| E484A crRNA (10th) 16-nt IVT template | AAACCTGCAACACCAT-ATCTACACTTAGTAGAAATTA-CCCTATAGTGAGTCGTATTAATTTC | Rejected | This study |
| E484A crRNA (12th) 16-nt | UAAUUUCUACUAAGUGUAGAU-UAAUGGUGUUGCAGGU | Rejected | This study |
| E484A crRNA (12th) 16-nt IVT template | ACCTGCAACACCATTA-ATCTACACTTAGTAGAAATTA-CCCTATAGTGAGTCGTATTAATTTC | Rejected | This study |
| E484K chimeric crRNA (-2nd) 24-nt | UAAUUUCUACUAAGUGUAGAU-ACACCAUUACAAGGUGTGCTACCG | Rejected | This study |
| E484K crRNA (-2nd) 24-nt | UAAUUUCUACUAAGUGUAGAU-ACACCAUUACAAGGUGUGCUACCG | Rejected | This study |
| E484K crRNA (-2nd) 24-nt IVT template | CGGTAGCACACCTTGTAATGGTGT-ATCTACACTTAGTAGAAATTA-CCCTATAGTGAGTCGTATTAATTTC | Rejected | This study |
| E484K crRNA (-2nd) 20-nt | UAAUUUCUACUAAGUGUAGAU-ACACCAUUACAAGGUGUGCU | Rejected | (4) |
| E484K crRNA (-2nd) 20-nt IVT template | AGCACACCTTGTAATGGTGT-ATCTACACTTAGTAGAAATTA-CCCTATAGTGAGTCGTATTAATTTC | Rejected | (4) |
| E484K crRNA (-2nd) 16-nt | UAAUUUCUACUAAGUGUAGAU-ACACCAUUACAAGGUG | Rejected | This study |
| E484K crRNA (-2nd) 16-nt IVT template | CACCTTGTAATGGTGT-ATCTACACTTAGTAGAAATTA-CCCTATAGTGAGTCGTATTAATTTC | Rejected | This study |
| E484K chimeric crRNA (6th) 24-nt | UAAUUUCUACUAAGUGUAGAU-GUGUUAAAGGUUUUAATTGTTACT | Rejected | This study |
| E484K crRNA (6th) 24-nt | UAAUUUCUACUAAGUGUAGAU-GUGUUAAAGGUUUUAAUUGUUACU | Rejected | This study |
| E484K crRNA (6th) 24-nt IVT template | AGTAACAATTAAAACCTTTAACAC-ATCTACACTTAGTAGAAATTA-CCCTATAGTGAGTCGTATTAATTTC | Rejected | This study |
| E484K crRNA (6th) 20-nt | UAAUUUCUACUAAGUGUAGAU-GUGUUAAAGGUUUUAAUUGU | Rejected | This study |
| E484K crRNA | ACAATTAAAACCTTTAACAC- | Rejected | This |

| | | | |
|---|---|---|---|
| (6th) 20-nt IVT template | ATCTACACTTAGTAGAAATTA-CCCTATAGTGAGTCGTATTAATTTC | | study |
| E484K crRNA (6th) 16-nt | UAAUUUCUACUAAGUGUAGAU-GUGUUAAAGGUUUUAA | Rejected | This study |
| E484K crRNA (6th) 16-nt IVT template | TTAAAACCTTTAACAC-ATCTACACTTAGTAGAAATTA-CCCTATAGTGAGTCGTATTAATTTC | Rejected | This study |
| E484K crRNA (9th) 16-nt | UAAUUUCUACUAAGUGUAGAU-AUGGUGUUAAAGGUUU | Rejected | This study |
| E484K crRNA (9th) 16-nt IVT template | AAACCTTTAACACCAT-ATCTACACTTAGTAGAAATTA-CCCTATAGTGAGTCGTATTAATTTC | Rejected | This study |
| E484K crRNA (9th) 15-nt | UAAUUUCUACUAAGUGUAGAU-AUGGUGUUAAAGGUU | Rejected | This study |
| E484K crRNA (9th) 15-nt IVT template | AACCTTTAACACCAT-ATCTACACTTAGTAGAAATTA-CCCTATAGTGAGTCGTATTAATTTC | Rejected | This study |
| E484K crRNA (11th) 16-nt | UAAUUUCUACUAAGUGUAGAU-UAAUGGUGUUAAAGGU | Rejected | This study |
| E484K crRNA (11th) 16-nt IVT template | ACCTTTAACACCATTA-ATCTACACTTAGTAGAAATTA-CCCTATAGTGAGTCGTATTAATTTC | Rejected | This study |
| E484Q chimeric crRNA (6th) 24-nt | UAAUUUCUACUAAGUGUAGAU-GUGUUCAAGGUUUUAA<u>TTGTTACT</u> | Rejected | This study |
| E484Q crRNA (6th) 24-nt | UAAUUUCUACUAAGUGUAGAU-GUGUUCAAGGUUUUAAUUGUUACU | Rejected | This study |
| E484Q crRNA (6th) 24-nt IVT template | AGTAACAATTAAAACCTTGAACAC-ATCTACACTTAGTAGAAATTA-CCCTATAGTGAGTCGTATTAATTTC | Rejected | This study |
| E484Q crRNA (6th) 20-nt | UAAUUUCUACUAAGUGUAGAU-GUGUUCAAGGUUUUAAUUGU | Rejected | This study |
| E484Q crRNA (6th) 20-nt IVT template | ACAATTAAAACCTTGAACAC-ATCTACACTTAGTAGAAATTA-CCCTATAGTGAGTCGTATTAATTTC | Rejected | This study |
| E484Q crRNA (6th) 16-nt | UAAUUUCUACUAAGUGUAGAU-GUGUUCAAGGUUUUAA | Rejected | This study |
| E484Q crRNA (6th) 16-nt IVT template | TTAAAACCTTGAACAC-ATCTACACTTAGTAGAAATTA-CCCTATAGTGAGTCGTATTAATTTC | Rejected | This study |
| E484Q crRNA (9th) 16-nt | UAAUUUCUACUAAGUGUAGAU-AUGGUGUUCAAGGUUU | Rejected | This study |
| E484Q crRNA (9th) 16-nt IVT template | AAACCTTGAACACCAT-ATCTACACTTAGTAGAAATTA-CCCTATAGTGAGTCGTATTAATTTC | Rejected | This study |
| E484Q crRNA (9th) 15-nt | UAAUUUCUACUAAGUGUAGAU-AUGGUGUUCAAGGUU | Rejected | This study |
| E484Q crRNA | AACCTTGAACACCAT- | Rejected | This |

| | | | |
|---|---|---|---|
| (9th) 15-nt IVT template | ATCTACACTTAGTAGAAATTA-CCCTATAGTGAGTCGTATTAATTTC | | study |
| E484Q crRNA (11th) 16-nt | UAAUUUCUACUAAGUGUAGAU-UAAUGGUGUUCAAGGU | Rejected | This study |
| E484Q crRNA (11th) 16-nt IVT template | ACCTTGAACACCATTA-ATCTACACTTAGTAGAAATTA-CCCTATAGTGAGTCGTATTAATTTC | Rejected | This study |
| F490S crRNA 18-nt | UAAUUUCUACUAAGUGUAGAU-AUUGUUACUCUCCUUUAC | Rejected | This study |
| F490S crRNA 18-nt IVT template | GTAAAGGAGAGTAACAAT-ATCTACACTTAGTAGAAATTA-CCCTATAGTGAGTCGTATTAATTTC | Rejected | This study |
| F490S crRNA 16-nt | UAAUUUCUACUAAGUGUAGAU-AUUGUUACUCUCCUUU | Rejected | This study |
| F490S crRNA 16-nt IVT template | AAAGGAGAGTAACAAT-ATCTACACTTAGTAGAAATTA-CCCTATAGTGAGTCGTATTAATTTC | Rejected | This study |
| F490S crRNA 15-nt | UAAUUUCUACUAAGUGUAGAU-AUUGUUACUCUCCUU | Rejected | This study |
| F490S crRNA 15-nt IVT template | AAGGAGAGTAACAAT-ATCTACACTTAGTAGAAATTA-CCCTATAGTGAGTCGTATTAATTTC | Rejected | This study |
| G493R+G496S+Q498R crRNA 18-nt | UAAUUUCUACUAAGUGUAGAU-CGAUCAUAUAGUUUCCGA | Rejected | This study |
| G493R+G496S+Q498R crRNA 18-nt IVT template | TCGGAAACTATATGATCG-ATCTACACTTAGTAGAAATTA-CCCTATAGTGAGTCGTATTAATTTC | Rejected | This study |
| G493R+G496S+Q498R crRNA 17-nt | UAAUUUCUACUAAGUGUAGAU-CGAUCAUAUAGUUUCCG | Rejected | This study |
| G493R+G496S+Q498R crRNA 17-nt IVT template | CGGAAACTATATGATCG-ATCTACACTTAGTAGAAATTA-CCCTATAGTGAGTCGTATTAATTTC | Rejected | This study |
| N501Y crRNA 24-nt | UAAUUUCUACUAAGUGUAGAU-CAACCCACUUAUGGUGUUGGUUAC | Rejected | (2) |
| N501Y crRNA 24-nt IVT template | GTAACCAACACCATAAGTGGGTTG-ATCTACACTTAGTAGAAATTA-CCCTATAGTGAGTCGTATTAATTTC | Rejected | (2) |
| N501Y crRNA 20-nt | UAAUUUCUACUAAGUGUAGAU-CAACCCACUUAUGGUGUUGG | Rejected | (2) |
| N501Y crRNA 20-nt IVT template | CCAACACCATAAGTGGGTTG-ATCTACACTTAGTAGAAATTA-CCCTATAGTGAGTCGTATTAATTTC | Rejected | (2) |
| N501Y crRNA 17-nt | UAAUUUCUACUAAGUGUAGAU-CAACCCACUUAUGGUGU | Rejected | This study |
| N501Y crRNA 17-nt IVT template | ACACCATAAGTGGGTTG-ATCTACACTTAGTAGAAATTA-CCCTATAGTGAGTCGTATTAATTTC | Rejected | This study |

| | | | |
|---|---|---|---|
| N501Y crRNA 16-nt | UAAUUUCUACUAAGUGUAGAU-CAACCCACUUAUGGUG | Rejected | This study |
| N501Y crRNA 16-nt IVT template | CACCATAAGTGGGTTG-ATCTACACTTAGTAGAAATTA-CCCTATAGTGAGTCGTATTAATTTC | Rejected | This study |

Selection criteria: the crRNA with the best specificity and reaction speed was selected for each mutation site, the others will be rejected. Introduced mismatches are in red font, and DNA sequences in the crRNAs are underlined. All templates mentioned above were purchased from Beijing Genomics Institute.

**Table S2 Details of clinical samples.**

| Sample No. | Accession No. (GISAID) | Variant type | Ct Value |
|---|---|---|---|
| 1 | EPI_ISL_6011981 | Alpha | 30.3 |
| 2 | EPI_ISL_2600360 | Beta | 20.9 |
| 3 | EPI_ISL_3066045 | WT | 28.1 |
| 4 | NTC | NTC | NTC |
| 5 | EPI_ISL_5937305 | Delta | 34.1 |
| 6 | NTC | NTC | NTC |
| 7 | EPI_ISL_8880070 | Omicron (BA.1) | 30.2 |
| 8 | EPI_ISL_5937311 | Alpha | 21.9 |
| 9 | EPI_ISL_4342620 | Delta | 21.3 |
| 10 | EPI_ISL_5937304 | Beta | 23.0 |
| 11 | NTC | NTC | NTC |
| 12 | EPI_ISL_8880074 | Omicron (BA.2) | 29.1 |
| 13 | NTC | NTC | NTC |
| 14 | EPI_ISL_6011980 | Alpha | 24.1 |
| 15 | EPI_ISL_9643325 | Omicron (BA.2) | 30.5 |
| 16 | EPI_ISL_3065973 | WT | 19.1 |
| 17 | EPI_ISL_5937317 | Alpha | 23.6 |
| 18 | EPI_ISL_9643312 | Omicron (BA.2) | 28.0 |
| 19 | NTC | NTC | NTC |
| 20 | EPI_ISL_4342636 | Delta | 17.1 |
| 21 | EPI_ISL_3065977 | WT | 17.2 |
| 22 | EPI_ISL_5937309 | Beta | 32.9 |
| 23 | NTC | NTC | NTC |
| 24 | EPI_ISL_4342618 | Delta | 19.8 |
| 25 | EPI_ISL_2484770 | Beta | 32.8 |
| 26 | EPI_ISL_4342638 | Delta | 19.0 |
| 27 | EPI_ISL_5937330 | Delta | 33.4 |
| 28 | EPI_ISL_8880072 | Omicron (BA.1) | 30.3 |
| 29 | EPI_ISL_4342630 | Delta | 33.3 |
| 30 | EPI_ISL_8880073 | Omicron (BA.1) | 27.1 |
| 31 | EPI_ISL_8880075 | Omicron (BA.2) | 27.9 |
| 32 | EPI_ISL_4342640 | Delta | 22.0 |
| 33 | EPI_ISL_4342641 | Delta | 30.3 |
| 34 | EPI_ISL_5937313 | Alpha | 20.7 |
| 35 | EPI_ISL_5937327 | Beta | 33.6 |
| 36 | EPI_ISL_4760501 | Delta | 30.8 |
| 37 | EPI_ISL_4760504 | Delta | 29.4 |
| 38 | EPI_ISL_8880069 | Omicron (BA.1) | 25.5 |
| 39 | EPI_ISL_4760503 | Delta | 29.8 |
| 40 | EPI_ISL_8880078 | Omicron (BA.2) | 31.8 |
| 41 | EPI_ISL_5937312 | Alpha | 27.3 |
| 42 | EPI_ISL_4342622 | Delta | 33.6 |
| 43 | EPI_ISL_4342623 | Delta | 31.3 |
| 44 | EPI_ISL_9643313 | Omicron (BA.2) | 25.9 |

| | | | |
|---|---|---|---|
| 45 | EPI_ISL_3065982 | WT | 21.1 |
| 46 | EPI_ISL_9643314 | Omicron (BA.2) | 31.8 |
| 47 | EPI_ISL_9643324 | Omicron (BA.2) | 31.5 |
| 48 | EPI_ISL_9643320 | Omicron (BA.2) | 33.2 |
| 49 | EPI_ISL_4342643 | Delta | 27.6 |
| 50 | EPI_ISL_4342645 | Delta | 23.4 |
| 51 | EPI_ISL_4760500 | Delta | 30.2 |
| 52 | EPI_ISL_5937314 | Alpha | 24.4 |
| 53 | EPI_ISL_2484791 | Beta | 33.7 |
| 54 | EPI_ISL_4760502 | Delta | 29.7 |
| 55 | EPI_ISL_5937308 | Delta | 31.1 |
| 56 | EPI_ISL_5937307 | Delta | 34.6 |
| 57 | EPI_ISL_3065989 | WT | 16.3 |
| 58 | EPI_ISL_8880071 | Omicron (BA.1) | 28.9 |
| 59 | EPI_ISL_8880076 | Omicron (BA.2) | 30.4 |
| 60 | EPI_ISL_8880077 | Omicron (BA.2) | 27.6 |
| 61 | NTC | NTC | NTC |
| 62 | NTC | NTC | NTC |
| 63 | EPI_ISL_5937318 | Alpha | 28.7 |
| 64 | EPI_ISL_9643316 | Omicron (BA.2) | 34.9 |
| 65 | EPI_ISL_5937321 | Alpha | 20.3 |
| 66 | EPI_ISL_9643315 | Omicron (BA.2) | 27.0 |
| 67 | EPI_ISL_9643317 | Omicron (BA.2) | 29.3 |
| 68 | EPI_ISL_5937316 | Alpha | 28.7 |
| 69 | EPI_ISL_9643318 | Omicron (BA.2) | 31.3 |
| 70 | EPI_ISL_9643319 | Omicron (BA.2) | 33.8 |
| 71 | EPI_ISL_2600354 | Beta | 35.9 |
| 72 | EPI_ISL_9643321 | Omicron (BA.2) | 31.6 |
| 73 | EPI_ISL_9643322 | Omicron (BA.2) | 32.1 |
| 74 | EPI_ISL_9643323 | Omicron (BA.2) | 31.0 |
| 75 | NTC | NTC | NTC |
| 76 | EPI_ISL_5937329 | Beta | 34.2 |
| 77 | NTC | NTC | NTC |
| 78 | EPI_ISL_2484776 | Beta | 34.6 |
| 79 | EPI_ISL_2484799 | Beta | 35.4 |

NTC: no template control.

**Table S3 Cost of reagents used in the SAVED platform.**

| Reagent | Unit Cost (US$)/Quantity | Quantity for each reaction | Cost (US$) for each reaction | Company | Catalog Number |
|---|---|---|---|---|---|
| Reverse Transcriptase | 437.8/50,000 units | 200 units | 1.75 | Thermo Fisher | EP0442 |
| RNase H | 312.6/1,000 units | 1 unit | 0.31 | Ambion | AM2293 |
| RPA (liquid basic) | 228.8/250 reactions | 1 reaction | 0.92 | TwistDx | TALQBAS01 |
| LbCas12a | 250/2,000 pmol | 2 pmol | 0.25 | NEB | M0653T |
| Primers, crRNAs, Reporter, buffer | | | ~0 | | |
| Total | | | 3.23 | | |

Blue LED light resource and filter are non-consumable. The cost per reaction (US$3.23) is comparable to RT-qPCR (US$2.74, TaqPath™ 1-Step RT-qPCR Master Mix, CG; Thermo Fisher; Catalog number: A15299).

**Table S4 RT-RPA primers used in this study.**

| Name | Sequence | Note | REF |
|---|---|---|---|
| **Selected RT-RPA primers** | | | |
| Δ144 RPA F | GACCCAGTCCCTACTTATTGTTAATAACGC | Forward primer for D138Y, G142D, G142D+Δ143-145, Δ144, and YY144–145TSN | This study |
| Δ144 RPA R | AAAGTGCAATTATTCGCACTAGAATAAACTCTGAACTC | Reverse primer for D138Y, G142D, G142D+Δ143-145, Δ144, and YY144–145TSN | (4) |
| Δ242-244 RPA F | TTCGGCTTTAGAACCATTGGTAGATTTGCC | Forward primer for Δ242-244 and R246N+Δ247-253 | This study |
| Δ242-244 RPA R | CCCACATAATAAGCTGCAGCACCAGCTGTC | Reverse primer for Δ242-244 and R246N+Δ247-253 | This study |
| K417N/T RPA F | ATGAAGTCAGACAAATCGCTCCAGGGCAAACT | Degenerate primer for K417N and K417T introduced PAM primers | This study |
| K417N/T PAM RPA (7/6th) F | TCAGACAAATCGCTCCAGGGCTTTCTGGAA | Introduced PAM in the forward primer for K417N/T, SNP in the 7th/6th nt of the crRNA | This study |
| K417N/T RPA R | ATTCCAAGCTATAACGCAGCCTGTAAAATC | Reverse primer for K417N and K417T | This study |
| L452Q/R RPA F | CTTGATTCTAAGGTTGGTGGTAATTATAAT | Degenerate primer for L452Q and L452R introduced PAM primers | This study |
| L452Q/R PAM RPA (11th) F | CTTGATTCTAAGGTTGGTGGTTTTTATAAT | Introduced PAM in the forward primer for L452Q and L452R, SNP in the 11th nt of the crRNA, okay | This study |
| L452Q/R RPA R | AAGGTTTGAGATTAGACTTCCTAAACAATC | Reverse primer for L452Q and L452R | This study |
| T478K RPA F | TTGAGAGAGATATTTCAACTGAAATCTATC | Forward primer for T478K | This study |
| T478K RPA R | AGTGGGTTGGAAACCATATGATTGTAAAGG | Reverse primer for T478K | This study |
| E484A RPA F | CAGGCCGGTAACAAACCTTGTAATGGTGTT | Degenerate primer for E484A introduced PAM primer; Forward primer for G493R+G496S | This study |
| E484A PAM RPA (12th) F | ATCAGGCCGGTAACAAACTTTGTAATGGTG | Introduced PAM in the forward primer for E484A, SNP in the 12th nt of the crRNA | This study |
| N501Y RPA F | CAGGCCGGTAGCACACCTTGTAATGGTG | Degenerate primer for E484K and E484Q introduced PAM | (2) |

| | | | |
|---|---|---|---|
| | TT | primers; Forward primer for F490S and N501Y | |
| E484K/Q PAM RPA (11th) F | ATCAGGCCGGTAGCACACTTTGTAATGGTG | Introduced PAM in the forward primer for E484K and E484Q, SNP in the 11th nt of the crRNA | This study |
| N501Y RPA R | TTGCTGGTGCATGTAGAAGTTCAAAAGAAAG | Reverse primer for: E484A/K/Q, F490S, G493R+G496S, and N501Y | (2) |
| N2 RPA F | GGGACCAGGAACTAATCAGACAAGGAACTG | Forward primer for N2 | This study |
| N2 RPA R | CGTTCCCGAAGGTGTGACTTCCATGCCAATGC | Reverse primer for N2 | This study |
| **Additional RT-RPA primers** | | | |
| T95I RPA F1 | GGTTTGATAACCCTGTCCTACCATTTAATG | Rejected | This study |
| T95I RPA F2 | TAACCCTGTCCTACCATTTAATGATGGTG | Rejected | This study |
| T95I RPA F3 | TAACCCTGTCCTACCATTTAATGATGGTGTTTATTTT | Rejected | This study |
| T95I RPA R1 | GGGACTGGGTCTTCGAATCTAAAGTAGTACC | Rejected | This study |
| T95I RPA R2 | TATTAACAATAAGTAGGGACTGGGTCTTCG | Rejected | This study |
| T95I RPA R3 | GCGTTATTAACAATAAGTAGGGACTGGGTCTTCGAAT | Rejected | This study |
| Δ144 RPA F1 | ACGCTACTAATGTTGTTATTAAAGTCTGTG | Rejected | This study |
| Δ144 RPA R1 | TAGAATAAACTCTGAACTCACTTTCCATCC | Rejected | This study |
| Δ144 RPA R2 | AAGTGCAATTATTCGCACTAGAATAAACTC | Rejected | This study |
| Δ144 RPA R3 | AGAGACATATTCAAAAGTGCAATTATTCGC | Rejected | This study |
| R190S RPA F1 | AATAATTGCACTTTTGAATATGTCTCTCAGCC | Rejected | This study |
| R190S RPA | CTTATGGACCTTGA | Rejected | This |

| | | | |
|---|---|---|---|
| F2 | AGGAAAACAGGGT AAT | | study |
| R190S RPA F3 | GTCTCTCAGCCTTT TCTTATGGACCTTG AAG | Rejected | This study |
| R190S RPA R1 | ATAACCATCAATATT CTTAAACACAAATT C | Rejected | This study |
| R190S RPA R2 | GGTTCTAAAGCCGA AAAACCCTGAGGG AGA | Rejected | This study |
| R190S RPA R3 | CACGCACTAAATTA ATAGGCGTGTGCTT AG | Rejected | This study |
| R190S RPA R4 | CCCTGAGGGAGATC ACGCACTAAATTAA TAGG | Rejected | This study |
| R190S RPA R5 | TCTACCAATGGTTCT AAAGCCGAAAAAC CC | Rejected | This study |
| Δ242-244 RPA F1 | CATTGGTAGATTTG CCAATAGGTATTAA C | Rejected | This study |
| Δ242-244 RPA F2 | GTGGTCTCCCTCAG GGTTTTTCGGCTTT AG | Rejected | This study |
| Δ242-244 RPA F3 | GATTTGCCAATAGG TATTAACATCACTAG G | Rejected | This study |
| Δ242-244 RPA R1 | GCTGTCCAACCTGA AGAAGAATCACCAG GA | Rejected | This study |
| Δ242-244 RPA R2 | GTCCTAGGTTGAAG ATAACCCACATAATA AG | Rejected | This study |
| Δ242-244 RPA R3 | GCAGCACCAGCTGT CCAACCTGAAGAA GAATC | Rejected | This study |
| R346K RPA F1 | TAGAGTCCAACCAA CAGAATCTATTGTTA G | Rejected | This study |
| R346K RPA F2 | GTTAGATTTCCTAAT ATTACAAACTTGTG C | Rejected | This study |
| R346K RPA F3 | GTTAGATTTCCTAAT ATTACAAACTTGTG CCC | Rejected | This study |
| R346K RPA R1 | ACAGTTGCTGATTC TCTTCCTGTTCCAA | Rejected | This study |

| | | | |
|---|---|---|---|
| | GC | | |
| R346K RPA R2 | TAATCAGCAACACAGTTGCTGATTCTCTTCC | Rejected | This study |
| R346K RPA R3 | GTTGCTGATTCTCTTCCTGTTCCAAGCATAAAC | Rejected | This study |
| K417N/T RPA F1 | AAGTCAGACAAATCGCTCCAGGGCAAACTG | Rejected | This study |
| K417N PAM RPA (7th) F1 | TCAGACAAATCGCTCCAGGGCTTTCTGGA | Rejected | This study |
| K417N PAM RPA (7th) F2 | TCAGACAAATCGCTCCAGGGCTTTCTGG | Rejected | This study |
| K417N PAM RPA (7th) F3 | ATGAAGTCAGACAAATCGCTCCAGGGCTTTCT | Rejected | This study |
| K417N PAM RPA (7th) F4 | ATGAAGTCAGACAAATCGCTCCAGGGCTTTCTGG | Rejected | This study |
| K417T PAM RPA (10th) F1 | GAAGTCAGACAAATCGCTCCATTTCAAACT | Rejected | This study |
| K417T PAM RPA (10th) F2 | TCAGACAAATCGCTCCATTTCAAACTGGAA | Rejected | This study |
| K417T PAM RPA (10th) F3 | TCAGACAAATCGCTCCATTTCAAACTGG | Rejected | This study |
| K417T PAM RPA (10th) F4 | ATGAAGTCAGACAAATCGCTCCATTTCAAACT | Rejected | This study |
| K417N/T RPA F1 | TAAATGATCTCTGCTTTACTAATGTCTATGC | Rejected | This study |
| K417N/T RPA F2 | TATGCAGATTCATTTGTAATTAGAGGTGATGAA | Rejected | This study |
| K417N/T RPA F3 | ATTTGTAATTAGAGGTGATGAAGTCAGACA | Rejected | This study |
| K417N/T RPA R1 | CAAGATTGTTAGAATTCCAAGCTATAACGCAG | Rejected | This study |
| K417N/T RPA R2 | TTGTTAGAATTCCAAGCTATAACGCAGCC | Rejected | This study |
| K417N/T | CTATAACGCAGCCT | Rejected | This |

| | | | |
|---|---|---|---|
| RPA R3 | GTAAAATCATCTGGTA | | study |
| L452Q/R RPA F1 | GATTCTAAGGTTGGTGGTAATTATAATTAC | Rejected | This study |
| L452Q/R RPA F2 | CTTGGAATTCTAACAATCTTGATTCTAAGGTT | Rejected | This study |
| L452Q/R RPA F3 | GAATTCTAACAATCTTGATTCTAAGGTTGGTG | Rejected | This study |
| L452Q/R RPA F4 | TCTAACAATCTTGATTCTAAGGTTGGTGGTAA | Rejected | This study |
| L452Q/R PAM RPA (7th) F1 | ATTCTAAGGTTGGTGGTAATTTTAATTACC | Rejected | This study |
| L452Q/R PAM RPA (7th) F2 | ATTCTAAGGTTGGTGGTAATTTTAATTAC | Rejected | This study |
| L452Q/R PAM RPA (7th) F3 | ATTCTAAGGTTGGTGGTAATTTTAATTA | Rejected | This study |
| L452Q/R PAM RPA (9th) F1 | ATTCTAAGGTTGGTGGTATTTATAATTACC | Rejected | This study |
| L452Q/R PAM RPA (9th) F2 | TCTTGATTCTAAGGTTGGTGGTATTTATAAT | Rejected | This study |
| L452Q/R PAM RPA (10/11th) F1 | ATTCTAAGGTTGGTGGTTTTTATAATTACC | Rejected | This study |
| L452Q/R PAM RPA (10/11th) F2 | TCTTGATTCTAAGGTTGGTGGTTTTTATAAT | Rejected | This study |
| L452Q/R PAM RPA (12th) F | ATTCTAAGGTTGGTGTTTATTATAATTACC | Rejected | This study |
| L452Q/R RPA R1 | TTCAGTTGAAATATCTCTCTCAAAAGGTTTG | Rejected | This study |
| L452Q/R RPA R2 | TACCGGCCTGATAGATTTCAGTTGAAATATC | Rejected | This study |
| L452Q/R RPA R3 | ATAGATTTCAGTTGAAATATCTCTCTCAAAAGG | Rejected | This study |
| E484A PAM RPA (10th) F | ATCAGGCCGGTAACAAACCTTTTAATGG | Rejected | This study |

| | | | |
|---|---|---|---|
| | TG | | |
| E484K/Q PAM RPA (6th) F | CAGGCCGGTAGCACACCTTGTTTTGGTGTT | Rejected | This study |
| E484K/Q PAM RPA (9th) F | CAGGCCGGTAGCACACCTTTTAATGGTGTT | Rejected | This study |
| E484K RPA F1 | GTATAGATTGTTTAGGAAGTCTAATCTCAAACC | Rejected | This study |
| E484K RPA F2 | AAACCTTTTGAGAGAGATATTTCAACTGAA | Rejected | This study |
| E484K RPA R | GATTGTAAAGGAAAGTAACAATTAAAACC | Rejected | This study |
| N501Y RPA F1 | GGTGTTGAAGGTTTTAATTGTTACTTTCC | Rejected | This study |
| N501Y RPA R1 | GTAGAAGTTCAAAAGAAAGTACTACTACTCT | Rejected | This study |
| N501Y RPA R2 | CAAAAGAAAGTACTACTACTCTGTATGGTTG | Rejected | This study |
| N2 RPA F1 | AGACAAGGAACTGATTACAAACATTGGC | Rejected | This study |
| N2 RPA F2 | TTTTGGGGACCAGGAACTAATCAGACAAGG | Rejected | This study |
| N2 RPA R1 | CCTGTGTAGGTCAACCACGTTCCCGAAG | Rejected | This study |
| N2 RPA R2 | TCCCGAAGGTGTGACTTCCATGCCAATG | Rejected | This study |

Selection criteria: the primer set with the most robust band or fluorescence signal was selected for further study, the others will be rejected. Introduced mismatches are in red font. All primers mentioned above were purchased from Beijing Genomics Institute.

**Table S5 Synthetic gene fragments of SARS-CoV-2.**

| Gene name | Sequences with T7 promoter (for *in vitro* transcription, underlined) | Note |
|---|---|---|
| N2 | GAAATTAATACGACTCACTATAGGGCGGCAAAAACGTACTGCCACTAAAGCATACAATGTAACACAAGCTTTCGGCAGACGTGGTCCAGAACAAACCCAAGGAAATTTTGGGGACCAGGAACTAATCAGACAAGGAACTGATTACAAACATTGGCCGCAAATTGCACAATTTGCCCCCAGCGCTTCAGCGTTCTTCGGAATGTCGCGCATTGGCATGGAAGTCACACCTTCGGGAACGTGGTTGACCTACACAGGTGCCATCAAATTGGATGACAAAGATCCAAATTTCAAAGATCAAGTCATTTTGCTGAATAAGCATATTGA | Wuhan-Hu-1 (GenBank accession number: NC_045512.2) (5) |
| NTD wild type includes wild types of T95, D138, G142, V143, Y144, Y145, R190, LAL242-244, R246N+Δ247-253 | GAAATTAATACGACTCACTATAGGGATGTTTGTTTTTCTTGTTTTATTGCCACTAGTCTCTAGTCAGTGTGTTAATCTTACAACCAGAACTCAATTACCCCCTGCATACACTAATTCTTTCACACGTGGTGTTTATTACCCTGACAAAGTTTTCAGATCCTCAGTTTTACATTCAACTCAGGACTTGTTCTTACCTTTCTTTTCCAATGTTACTTGGTTCCATGCTATACATGTCTCTGGGACCAATGGTACTAAGAGGTTTGATAACCCTGTCCTACCATTTAATGATGGTGTTTATTTTGCTTCCACTGAGAAGTCTAACATAATAAGAGGCTGGATTTTTGGTACTACTTTAGATTCGAAGACCCAGTCCCTACTTATTGTTAATAACGCTACTAATGTTGTTATTAAAGTCTGTGAATTTCAATTTTGTAATGATCCATTTTTGGGTGTTTATTACCACAAAAACAACAAAAGTTGGATGGAAAGTGAGTTCAGAGTTTATTCTAGTGCGAATAATTGCACTTTTGAATATGTCTCTCAGCCTTTTCTTATGGACCTTGAAGGAAAACAGGGTAATTTCAAAAATCTTAGGGAATTTGTGTTTAAGAATATTGATGGTTATTTTAAAATATATTCTAAGCACACGCCTATTAATTTAGTGCGTGATCTCCCTCAGGGTTTTTCGGCTTTAGAACCATTGGTAGATTTGCCAATAGGTATTAACATCACTAGGTTTCAAACTTTACTTGCTTTACATAGAAGTTATTTGACTCCTGGTGATTCTTCTTCAGGTTGGACAGCTGGTGCTGCAGCTTATTATGTGGGTTATCTTCAACCTAGGACTTTTCTATTAAAATATAATGAAAATGGAACCATTACAGATGCTGTAGACTGTGCACTTGACCCTCTCTCAGAAACAAAGTGTACGTTGAAATCCTTCACTGTAGAAAAAGGAATCTATCAAACTTCTAACTTT | Wuhan-Hu-1 (GenBank accession number: NC_045512.2) (5) |

| | | |
|---|---|---|
| RBD wild type includes wild types of R346, K417, L452, T478, E484, F490, G493, G496, Q498, N501 | GAAATTAATACGACTCACTATAGGGAGAGTCCAACCAACAGAATCTATTGTTAGATTTCCTAATATTACAAACTTGTGCCCTTTTGGTGAAGTTTTTAACGCCACCAGATTTGCATCTGTTTATGCTTGGAACAGGAAGAGAATCAGCAACTGTGTTGCTGATTATTCTGTCCTATATAATTCCGCATCATTTTCCACTTTTAAGTGTTATGGAGTGTCTCCTACTAAATTAAATGATCTCTGCTTTACTAATGTCTATGCAGATTCATTTGTAATTAGAGGTGATGAAGTCAGACAAATCGCTCCAGGGCAAACTGGAAAGATTGCTGATTATAATTATAAATTACCAGATGATTTTACAGGCTGCGTTATAGCTTGGAATTCTAACAATCTTGATTCTAAGGTTGGTGGTAATTATAATTACCTGTATAGATTGTTTAGGAAGTCTAATCTCAAACCTTTTGAGAGAGATATTTCAACTGAAATCTATCAGGCCGGTAGCACACCTTGTAATGGTGTTGAAGGTTTTAATTGTTACTTTCCTTTACAATCATATGGTTTCCAACCCACTAATGGTGTTGGTTACCAACCATACAGAGTAGTAGTACTTTCTTTTGAACTTCTACATGCACCAGCAACTGTTTGTGGACCTAAAAAGTCTACTAATTTGGTTAAAAACAAATGTGTCAATTTC | Wuhan-Hu-1 (GenBank accession number: NC_045512.2) (5) |
| T95I, YY144–145TSN mutant | GAAATTAATACGACTCACTATAGGGCTCTGGGACCAATGGTACTAAGAGGTTTGATAACCCTGTCCTACCATTTAATGATGGTGTTTATTTTGCTTCCATTGAGAAGTCTAACATAATAAGAGGCTGGATTTTTGGTACTACTTTAGATTCGAAGACCCAGTCCCTACTTATTGTTAATAACGCTACTAATGTTGTTATTAAAGTCTGTGAATTTCAATTTTGTAATGATCCATTTTTGGGTGTTACTTCTAACCACAAAAACAACAAAAGTTGGATGGAAAGTGAGTTCAGAGTTTATTCTAGTGCGAATAATTGCACTTTTGAATATGTCTCT | Mu variant (GISAID accession ID: EPI_ISL_4036743) (6) |
| D138Y, R190S mutant | GAAATTAATACGACTCACTATAGGGTAGATTCGAAGACCCAGTCCCTACTTATTGTTAATAACGCTACTAATGTTGTTATTAAAGTCTGTGAATTTCAATTTTGTAATTATCCATTTTTGGGTGTTTATTACCACAAAAACAACAAAAGTTGGATGGAAAGTGAGTTCAGAGTTTATTCTAGTGCGAATAATTGCACTTTTGAATATGTCTCTCAGCCTTTTCTTATGGACCTTGAAGGAAAACAGGGTAATTTCAAAAATCTTAGTGAATTTGTGTTTAAGAATATTGATGGTTATTTTAAAATATATTCTAAGCACACGCCTATTAATTTAGTGCGTGATCTCCCTCAGGGTTTTTCGGCTTTAGAACCATTGGTAGA | Gamma variant (GISAID accession ID: EPI_ISL_3695801) (6) |
| G142D | GAAATTAATACGACTCACTATAGGGTAGATTCGAAGACCCAGTCCCTACTTATTGTTAATAACGCTACTAATGTTGTTATTAAAGTCTGTGAATTTCAATTTTGTAATGATCCATTTTTGGATGTTTATTACCACAAAAACAACAAAAGTTGGATGGAAAGTGAGTTCAGAGTTTATTCTAGTGCGAATAATTGCACTTTTGAATATGTCTCT | Omicron BA.2 subvariant (GISAID accession ID: EPI_ISL_913432 |

| | | |
|---|---|---|
| | | 0) (6) |
| G142D+ Δ143-145 mutant | GAAATTAATACGACTCACTATAGGGTAGATTCGAAGACCCAGTCCCTACTTATTGTTAATAACGCTACTAATGTTGTTATTAAAGTCTGTGAATTTCAATTTTGTAATGATCCATTTTTGGACCACAAAAACAACAAAAGTTGGATGGAAAGTGAGTTCAGAGTTTATTCTAGTGCGAATAATTGCACTTTTGAATATGTCTCT | Omicron BA.1 subvariant (GISAID accession ID: EPI_ISL_6640919) (6) |
| Δ144 mutant | GAAATTAATACGACTCACTATAGGGTAGATTCGAAGACCCAGTCCCTACTTATTGTTAATAACGCTACTAATGTTGTTATTAAAGTCTGTGAATTTCAATTTTGTAATGATCCATTTTTGGGTGTTTACCACAAAAACAACAAAAGTTGGATGGAAAGTGAGTTCAGAGTTTATTCTAGTGCGAATAATTGCACTTTTGAATATGTCTCT | Alpha variant (GISAID accession ID: EPI_ISL_3695798) (6) |
| D215G, Δ242-244 mutant | GAAATTAATACGACTCACTATAGGGTAGTGCGTGGTCTCCCTCAGGGTTTTTCGGCTTTAGAACCATTGGTAGATTTGCCAATAGGTATTAACATCACTAGGTTTCAAACTTTACATAGAAGTTATTTGACTCCTGGTGATTCTTCTTCAGGTTGGACAGCTGGTGCTGCAGCTTATTATGTGGGTTATCTTCAACCTAGGACTTTTCTATT | Beta variant (GISAID accession ID: EPI_ISL_3692589) (6) |
| R246N+ Δ247-253 mutant | GAAATTAATACGACTCACTATAGGGTAGTGCGTGATCTCCCTCAGGGTTTTTCGGCTTTAGAACCATTGGTAGATTTGCCAATAGGTATTAACATCACTAGGTTTCAAACTTTACTTGCTTTACATAATTCTTCTTCAGGTTGGACAGCTGGTGCTGCAGCTTATTATGTGGGTTATCTTCAACCTAGGACTTTTCTATT | Lambda variant (GISAID accession ID: EPI_ISL_3050714) (6) |
| R346K mutant | GAAATTAATACGACTCACTATAGGGTAGAGTCCAACCAACAGAATCTATTGTTAGATTTCCTAATATTACAAACTTGTGCCCTTTTGGTGAAGTTTTTAACGCCACCAAATTTGCATCTGTTTATGCTTGGAACAGGAAGAGAATCAGCAACTGTGTTGCTGATTATTCTGTCCTATATAATTCCGCA | Mu variant (GISAID accession ID: EPI_ISL_4036743) (6) |

| | | |
|---|---|---|
| K417N, E484K, N501Y mutant | GAAATTAATACGACTCACTATAGGGTGTTATGGAGT<br>GTCTCCTACTAAATTAAATGATCTCTGCTTTACTAAT<br>GTCTATGCAGATTCATTTGTAATTAGAGGTGATGAA<br>GTCAGACAAATCGCTCCAGGGCAAACTGGAAATAT<br>TGCTGATTATAATTATAAATTACCAGATGATTTTACA<br>GGCTGCGTTATAGCTTGGAATTCTAACAATCTTGAT<br>TCTAAGGTTGGTGGTAATTATAATTACCTGTATAGAT<br>TGTTTAGGAAGTCTAATCTCAAACCTTTTGAGAGA<br>GATATTTCAACTGAAATCTATCAGGCCGGTAGCACA<br>CCTTGTAATGGTGTTAAAGGTTTTAATTGTTACTTT<br>CCTTTACAATCATATGGTTTCCAACCCACTTATGGT<br>GTTGGTTACCAACCATACAGAGTAGTAGTACTTTCT<br>TTTGAACTTCTACATGCACCAGCAACTGTTTGTGG<br>ACCTAAAAAGTCTACTAATTTGGTTAAAAACAAAT<br>GTGTCAATTTC | Beta variant (GISAID accession ID: EPI_ISL_3692589) (6) |
| K417T mutant | GAAATTAATACGACTCACTATAGGGTGTTATGGAGT<br>GTCTCCTACTAAATTAAATGATCTCTGCTTTACTAAT<br>GTCTATGCAGATTCATTTGTAATTAGAGGTGATGAA<br>GTCAGACAAATCGCTCCAGGGCAAACTGGAACGA<br>TTGCTGATTATAATTATAAATTACCAGATGATTTTAC<br>AGGCTGCGTTATAGCTTGGAATTCTAACAATCTTGA<br>TTCTAAGGTTGGTGGTAATTATAA | Gamma variant (GISAID accession ID: EPI_ISL_3695801) (6) |
| L452Q, F490S mutant | GAAATTAATACGACTCACTATAGGGTACCAGATGAT<br>TTTACAGGCTGCGTTATAGCTTGGAATTCTAACAAT<br>CTTGATTCTAAGGTTGGTGGTAATTATAATTACCAGT<br>ATAGATTGTTTAGGAAGTCTAATCTCAAACCTTTTG<br>AGAGAGATATTTCAACTGAAATCTATCAGGCCGGT<br>AGCACACCTTGTAATGGTGTTGAAGGTTTTAATTGT<br>TACTCTCCTTTACAATCATATGGTTTCCAACCCACT<br>AATGGTGTTGGTTACCAACCATACAGAGTAGTAGT<br>ACTTTCTTTTGAACTTCTACATGCACCAGCAACTGT<br>TTGTGGACCTAAAAAGTCTACTAATTTGGTTAAAA<br>ACAAATGTGTCAATTTC | Lambda variant (GISAID accession ID: EPI_ISL_3050714) (6) |
| L452R, T478K mutant | GAAATTAATACGACTCACTATAGGGTACCAGATGAT<br>TTTACAGGCTGCGTTATAGCTTGGAATTCTAACAAT<br>CTTGATTCTAAGGTTGGTGGTAATTATAATTACCGGT<br>ATAGATTGTTTAGGAAGTCTAATCTCAAACCTTTTG<br>AGAGAGATATTTCAACTGAAATCTATCAGGCCGGT<br>AGCAAACCTTGTAATGGTGTTGAAGGTTTTAATTGT<br>TACTTTCCTTTACAATCATATGGTTTCCAACCCACTA<br>ATGGTGTTGGTTACCAACCATACAGAGTAGTAGTA<br>CTTTCTTTTGAACTTCTACATGCACCAGCAACTGTT<br>TGTGGACCTAAAAAGTCTACTAATTTGGTTAAAAA<br>CAAATGTGTCAATTTC | Delta variant (GISAID accession ID: EPI_ISL_2922993) (6) |

| | | |
|---|---|---|
| E484Q | GAAATTAATACGACTCACTATAGGGGTATAGATTGTTTAGGAAGTCTAATCTCAAACCTTTTGAGAGAGATATTTCAACTGAAATCTATCAGGCCGGTAGCACACCTTGTAATGGTGTTCAAGGTTTTAATTGTTACTTTCCTTTACAATCATATGGTTTCCAACCCACTAATGGTGTTGGTTACCAACCATACAGAGTAGTAGTACTTTCTTTTGAACTTCTACATGCACCAGCAACTGTTTGTGGACCTAAAAAGTCTACTAATTTGGTTAAAAACAAATGTGTCAATTTC | Kappa variant (GISAID accession ID: EPI_ISL_3665668) (6) |
| S477N, T478K, E484A, Q493R, G496S, Q498R, N501Y, Y505H | GAAATTAATACGACTCACTATAGGGGTATAGATTGTTTAGGAAGTCTAATCTCAAACCTTTTGAGAGAGATATTTCAACTGAAATCTATCAGGCCGGTAACAAACCTTGTAATGGTGTTGCAGGTTTTAATTGTTACTTTCCTTTACGATCATATAGTTTCCGACCCACTTATGGTGTTGGTCACCAACCATACAGAGTAGTAGTACTTTCTTTTGAACTTCTACATGCACCAGCAACTGTTTGTGGACCTAAAAAGTCTACTAATTTGGTTAAAAACAAATGTGTCAATTTC | Omicron BA.1 subvariant (GISAID accession ID: EPI_ISL_6640919) (6) |
| S477N, T478K, E484A, Q493R, Q498R, N501Y, Y505H | GAAATTAATACGACTCACTATAGGGGTATAGATTGTTTAGGAAGTCTAATCTCAAACCTTTTGAGAGAGATATTTCAACTGAAATCTATCAGGCCGGTAACAAACCTTGTAATGGTGTTGCAGGTTTTAATTGTTACTTTCCTTTACGATCATATGGTTTCCGACCCACTTATGGTGTTGGTCACCAACCATACAGAGTAGTAGTACTTTCTTTTGAACTTCTACATGCACCAGCAACTGTTTGTGGACCTAAAAAGTCTACTAATTTGGTTAAAAACAAATGTGTCAATTTC | Omicron BA.2 subvariant (GISAID accession ID: EPI_ISL_9134320) (6) |

All gene fragments mentioned above were purchased from Beijing Genomics Institute.

## REFERENCES

1. Kellner MJ, Koob JG, Gootenberg JS, Abudayyeh OO, Zhang F. 2019. SHERLOCK: nucleic acid detection with CRISPR nucleases. Nat Protoc 14:2986–3012.
2. Yang J, Barua N, Rahman MN, Lo N, Tsang TF, Yang X, Chan PKS, Zhang L, Ip M. 2021. Chimeric crRNA improves CRISPR-Cas12a specificity in the N501Y mutation detection of Alpha, Beta, Gamma, and Mu variants of SARS-CoV-2. PLoS One 16:1–7.
3. Broughton JP, Deng X, Yu G, Fasching CL, Servellita V, Singh J, Miao X, Streithorst JA, Granados A, Sotomayor-Gonzalez A, Zorn K, Gopez A, Hsu E, Gu W, Miller S, Pan CY, Guevara H, Wadford DA, Chen JS, Chiu CY. 2020. CRISPR–Cas12-based detection of SARS-CoV-2. Nat Biotechnol 38:870–874.
4. de Puig H, Lee RA, Najjar. 2021. Minimally instrumented SHERLOCK (miSHERLOCK) for CRISPR-based point-of-care diagnosis of SARS-CoV-2 and emerging variants. Sci Adv 7.
5. SARS-CoV-2 wild-type NC_045512.2. https://www.ncbi.nlm.nih.gov/nuccore/NC_045512.
6. GISAID. https://gisaid.org/.
